# Accelerated simulation methodologies for computational vascular flow modelling

**DOI:** 10.1098/rsif.2023.0565

**Published:** 2024-02-14

**Authors:** Michael MacRaild, Ali Sarrami-Foroushani, Toni Lassila, Alejandro F. Frangi

**Affiliations:** ^1^ Centre for Computational Imaging and Simulation Technologies in Biomedicine (CISTIB), University of Leeds, Leeds, UK; ^2^ EPSRC Centre for Doctoral Training in Fluid Dynamics, University of Leeds, Leeds, UK; ^3^ School of Computing, University of Leeds, Leeds, UK; ^4^ School of Computer Science, University of Manchester, Manchester, UK; ^5^ School of Health Science, University of Manchester, Manchester, UK; ^6^ Department of Cardiovascular Sciences, KU Leuven, Leuven, Belgium; ^7^ Department of Electrical Engineering (ESAT), KU Leuven, Leuven, Belgium

**Keywords:** simulation acceleration, reduced order modelling, machine learning, vascular flow modelling, haemodynamics

## Abstract

Vascular flow modelling can improve our understanding of vascular pathologies and aid in developing safe and effective medical devices. Vascular flow models typically involve solving the nonlinear Navier–Stokes equations in complex anatomies and using physiological boundary conditions, often presenting a multi-physics and multi-scale computational problem to be solved. This leads to highly complex and expensive models that require excessive computational time. This review explores accelerated simulation methodologies, specifically focusing on computational vascular flow modelling. We review reduced order modelling (ROM) techniques like zero-/one-dimensional and modal decomposition-based ROMs and machine learning (ML) methods including ML-augmented ROMs, ML-based ROMs and physics-informed ML models. We discuss the applicability of each method to vascular flow acceleration and the effectiveness of the method in addressing domain-specific challenges. When available, we provide statistics on accuracy and speed-up factors for various applications related to vascular flow simulation acceleration. Our findings indicate that each type of model has strengths and limitations depending on the context. To accelerate real-world vascular flow problems, we propose future research on developing multi-scale acceleration methods capable of handling the significant geometric variability inherent to such problems.

## 1. Introduction

### 1.1. The motivation for accelerating vascular flow simulations

Despite the widespread use of computational models across many scientific disciplines, their use in real-time and many-query contexts is limited by their high computational cost. These scenarios frequently arise in vascular blood flow modelling. Real-time vascular flow simulations could provide guidance to clinicians prior to performing a treatment procedure or provide near-instant feedback during the procedure [1,2]. Many-query vascular flow simulations can be used to iteratively design new vascular implements, establish safety and performance measures for treatment devices, and simulate interventions on a population scale through so-called *in silico* trials [3].

### 1.2. Challenges in vascular flow modelling

**Figure 1.**
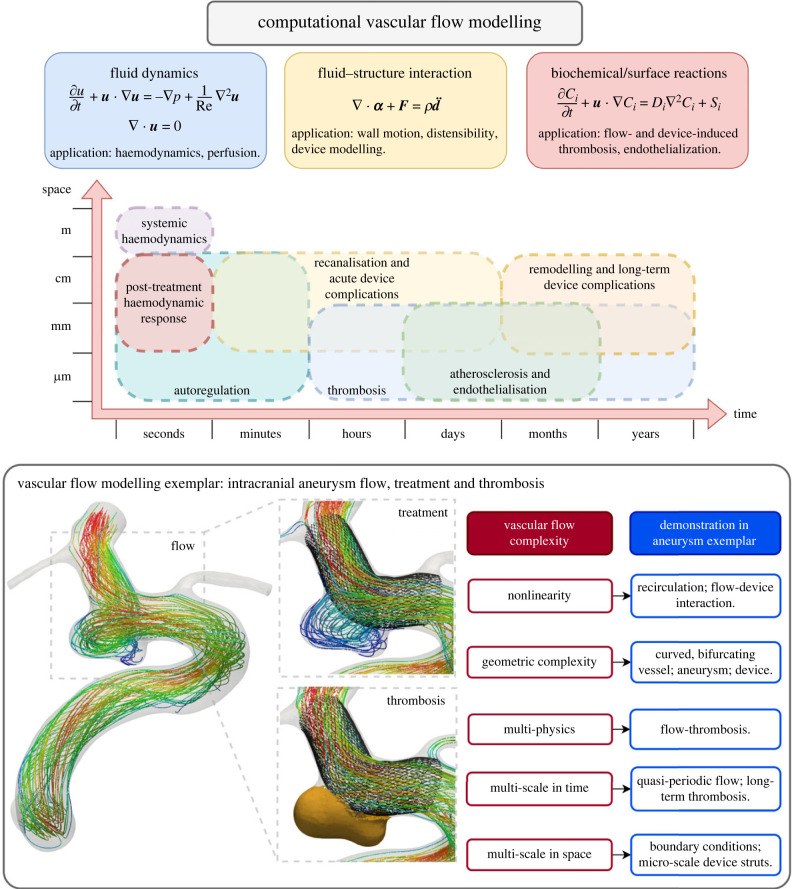
Vascular flow modelling is a multi-physics, multi-scale problem where nonlinearity and geometric complexity frequently arise.

Vascular flow modelling poses various challenges due to the inherent complexities of the problem, which are highlighted in figure 1 [4,5]. Blood flow dynamics and tissue perfusion are governed by the Navier–Stokes equations, which are a nonlinear set of time-dependent partial differential equations [6]. Coupling the haemodynamics to solid mechanics or biochemical reaction models may also be required in certain applications. Fluid–structure interaction (FSI) is required when vessel distensibility is important or when there is a complex interaction between blood flow and valves or implanted devices [7–10]. Biochemical reactions are crucial in modelling thrombosis and endothelialisation depends on interactions between blood and blood-contacting surfaces of devices [11,12]. The constitutive nature of blood adds additional complexity—it is a suspension containing various biochemically active particles and molecules, meaning that multi-phase multi-component flow-biochemistry models may be required when modelling flow-thrombosis in small vessels [11,13].

As well as being multi-physical in nature, the length and time scales in vascular flow problems can differ greatly. Vascular flow is inherently pulsatile, which leads to features such as flow separation, vortex transport, mixing regions and impingement varying topologically throughout the cardiac cycle [14]. Variation in length scale and morphology can also influence these flow features. This leads to varying flow regimes in different regions of the vasculature and at different times of the cardiac cycle. Vascular flow modelling encompasses short-term processes such as systemic haemodynamics, autoregulation and recanalisation in addition to long-term processes such as remodelling and thrombosis [15–18]. Physiological changes due to factors such as age and lifestyle also have an impact on various flow problems. Vastly different length scales are also present, with thrombosis and endothelialisation happening on a molecular level at the micro-scale, whereas systemic blood flow occurs in arteries with diameters up to a few centimetres.

Nonlinear effects further complicate vascular flow modelling. This can result from the convective nonlinearity in the Navier–Stokes equation, the geometric complexity of blood vessels, or the interactions across different length and time scales between blood flow and other physical and physiological phenomena. Nonlinear flow features are often found in the presence of vascular pathologies such as stenosis, atherosclerosis, aneurysms or valve defects [19–22]. Flow–device interactions can be an additional source of nonlinearity [23–25].

The most prominent complexities in vascular flow modelling can be summarised as: (i) nonlinearity, (ii) geometrical complexity, (iii) multi-physics, (iv) multi-scale in time, (v) multi-scale in space. In practice, assumptions can be made to simplify or eliminate these complexities for most problems, allowing for successful computational modelling. When aiming to accelerate vascular flow simulations, problem-specific approaches that are suited to handling particular types of complexity will be required depending upon the specific target application.

### 1.3. Reduced order models and machine learning for acceleration

Simulation acceleration refers to reducing the run time of computational models and is typically achieved through modelling assumptions and simplifications. Reduced order models (ROMs) are low-order representations of high-order models that preserve essential model input–output behaviour at the cost of some model accuracy and are a common approach for accelerating expensive computational models [26,27]. ROMs can be categorised into two families, *a priori ROMs* and *a posteriori ROMs*. The former seek to reduce the order of the system prior to solving the high-dimensional model, using techniques such as spatial dimension reduction (SDR) or proper generalised decomposition (PGD). *A posteriori ROMs* are data-driven techniques that depend on first solving the high-dimensional model or acquiring experimental data to generate snapshot solution fields. Snapshot data are decomposed into a reduced representation using, for example, proper orthogonal decomposition (POD) [28–31], dynamic mode decomposition (DMD) [32,33] or variants thereof. The reduced representation can then be advanced in time directly or combined with projection or interpolation techniques to construct a ROM. There are a multitude of ROM techniques, some of which have been applied to vascular flow problems.

Recent advances in machine learning have improved some ROM methodologies and provided alternative techniques to accelerate simulations. Machine learning acceleration methods operate under a similar paradigm to many ROM techniques, with an expensive offline training phase that primes the model for fast online inference in new geometries, parameter values or time points. There are various ways to use machine learning in simulation acceleration. Machine learning ROMs typically use machine learning to augment/replace a component of a ROM or they use machine learning entirely in place of existing ROM components [34,35]. Physics-informed machine learning strategies are another possibility. In this approach, flow measurements are supplemented by additional constraints based on the underlying governing equations and boundary conditions [36]. Physics-agnostic techniques ignore the underlying physics of the problem, but instead use large amounts of data to identify mappings from images or geometries to flow quantities of interest [37]. Other techniques include tailor-made networks designed to handle point-cloud data [38,39] and operator learning strategies [40,41]. Given the relatively recent emergence of machine learning simulation techniques, they have not been widely applied to acceleration of vascular flow simulations yet.

### 1.4. Overview

**Figure 2.**
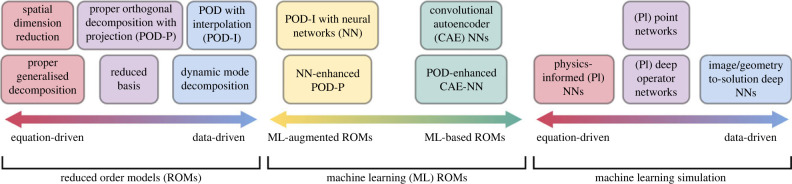
Taxonomy of various simulation acceleration methods reviewed in this paper.

This review aims to provide an overview of various methods for accelerating simulations (figure 2) and to collate, categorise, and critique each method with respect to the target application of vascular flow modelling. We decompose vascular flow modelling into a series of complexities (nonlinearity, geometric complexity, multi-physics and multi-scale in time and space) and assess various acceleration methods with respect to these complexities. For ROM approaches, we provide guidance on what type of vascular problems the method may be suitable for, what problems they have already been applied to, and how successful these studies were in terms of the accuracy and acceleration offered by the approach compared to traditional numerical methods. For machine learning approaches, we review some common methods, discuss their benefits and limitations, and advise what vascular problems they may be suitable for. Throughout this review, we measure acceleration factors by comparing run times for a single evaluation of the accelerated and full-order models (FOMs), unless otherwise stated. For complementary reviews on parametric model reduction, model order reduction in fluid dynamics, data-driven cardiovascular flow modelling, machine learning for cardiovascular biomechanics, real-time simulation of computational surgery, and the challenges of vascular fluid dynamics, see [5,26,29,42–44]. Finally, we note that although this review focuses on vascular flow acceleration, the complexities of this application (nonlinearity, geometric complexity, multi-physics and multi-scale) are encountered across many other computational modelling domains. Therefore, we believe this review will be useful to computational vascular flow modelling researchers and the broader computational modelling community.

## 2. Reduced order modelling of vascular flow

ROMs aim to reduce the dimensionality of a numerical problem either by applying prior knowledge of the problem itself or by inferring knowledge based on previously gathered data from the system of interest. ROM methods can be described as *a priori* or *a posteriori*, depending on whether the reduction of the system exploits prior knowledge about the FOM or information (data) collected after solving it, respectively. *A priori* methods are useful when there exist symmetries or other known information about the underlying system, or when the system is too complex to solve with traditional techniques. *A posteriori* methods are useful when readily available data from the FOM can be used to guide the construction of the ROM. Another categorisation for ROM methods is whether the approach is intrusive or non-intrusive. Intrusive methods require the explicit use of the underlying high-order numerical implementation of the FOM, whereas non-intrusive methods operate entirely separate to the FOM. Intrusive methods can be more numerically robust due to their incorporation of the underlying governing equations, but non-intrusive techniques can be easier to implement and use in conjunction with commercial solvers, which are common when studying fluid dynamics problems. Many categories of ROM have been applied to vascular flow, with various benefits and limitations to each approach. This section will describe some of the most common ROM techniques and their suitability to model various vascular flow complexities.

### 2.1. Spatial dimension reduction

The three-dimensional (3D) unsteady incompressible Navier–Stokes equations in non-dimensional form are: find $\left(u,p)\in H^{1}(\Omega ;R^{d})\times L^{2}(\Omega ;R\right)$ s.t.

2.1 $$\frac{\partial u}{\partial t}+u\cdot \nabla u=-\nabla p+\frac{1}{\text{Re}}\nabla^{2}u,\;\nabla \cdot u=0,$$

where ***u*** is the velocity, *p* is the pressure and Re is the Reynolds number dependent upon the fluid density *ρ* and dynamic viscosity *μ*. The spatial dimension is *d* = 3 except for some cases of plane symmetric or axisymmetric flow, when *d* = 2, and the domain $\Omega \subset R^{d}$ has a suitably regular boundary to ensure the existence of solutions. Spatial dimension reduction (SDR) involves reducing these equations down to a zero-dimensional (0D)/one-dimensional (1D)/two-dimensional (2D) model that describes bulk quantities instead of the full spatio-temporal flow fields. A comprehensive review of 0D and 1D techniques has been provided by Shi *et al.* [45]. We provide an overview of this approach, quantify the acceleration and accuracy offered, and discuss how applicable this method is to vascular flow simulation acceleration.

#### 2.1.1. Zero-dimensional models

Lumped parameter models (referred from hereon in as 0D models) exploit the analogy between hydraulic networks and electrical circuits. Blood pressure and flow rate are represented by voltage and current, and the frictional, inertial and elastic effects of blood flow are described by electrical resistance, inductance and capacitance, respectively [45]. Established methods for modelling electrical circuits (Kirchhoff’s current law, Ohm’s Law for voltage–current) with ordinary differential equations (ODEs) can then be used to describe vascular flow problems.

The first 0D models were based on the Windkessel model, which consists of a capacitor that describes the storage properties of large arteries and a resistor that describes the dissipative nature of small peripheral vessels [45]. This simple approach cannot model specific pressure and flow-rate changes in particular vascular segments and it cannot fully describe the effects of arterial impedance, venous pressure fluctuations, or pulse wave transmission. Various extensions to this model have been used to capture these more complex physiological phenomena by adding additional resistors, inductances, and capacitors. For example, in a system with capacitance/compliance *C*, voltage/pressure *P*, charge/flow rate *Q*, inductance/inertia *L* and resistance *R*, the two ODEs describing the system are [46]

2.2 $$C\frac{\text{d}P_{1}}{\text{d}t}+Q_{2}-Q_{1}=0\;\mathrm{and}\;L\frac{\text{d}Q_{2}}{\text{d}t}+P_{2}-P_{1}=-RQ_{2}.$$

Multi-compartment models can also be used to describe flow and pressure characteristics within specific vascular segments.

#### 2.1.2. One-dimensional models

In 1D models, the form of the velocity profile across the vessel radius is constrained, which simplifies the 3D governing equations. 1D blood flow is governed by the axisymmetric forms of the incompressible continuity and Navier–Stokes equations, which can be written as

2.3 $$\frac{\partial A}{\partial t}+\frac{\partial (AU)}{\partial x}=0\;\mathrm{and}\;\frac{\partial U}{\partial t}+U\frac{\partial U}{\partial x}+\frac{1}{\rho }\frac{\partial p}{\partial x}=\frac{\;f}{\rho A},$$

where *x* is a local coordinate describing the vessel segment, *A* is the cross-sectional area, *U* and *p* are the cross-sectionally averaged velocity and pressure, *ρ* is the blood density and *f* is a viscosity-dependent term describing the frictional force per unit length [45,47]. These equations can be further coupled to a pressure–radius relationship that describes the elasticity of the vessel wall. The reduced equations can be solved using various numerical techniques, such as the method of characteristics [48,49] or finite differences [50].

A primary benefit of 1D models over 0D models is that they can capture pressure and velocity pulse wave propagation [51]. Waves carry information about the medium in which they travel, so capturing the pressure and velocity waves in blood vessels can tell us about the function of the cardiovascular system and provide information about various vascular pathologies, such as atherosclerosis and hypertension [52].

#### 2.1.3. Two-dimensional models

For 2D vascular models, the 3D vessel loses its torsion and curvature, becoming a straightened tube governed by the 2D Navier–Stokes equations. 2D models include the radial variation of the velocity and pressure fields in an axisymmetric tube, whereas 1D models only consider the cross-sectionally averaged quantities. These models are used less frequently now due to improved computer processing power and widely available commercial solvers that make solving the 3D problem more tractable [53]. However, in certain applications, such as the calculation of fractional flow reserve (FFR), 2D models are shown to be significantly faster than 3D models while retaining a clinically viable level of accuracy [54].

#### 2.1.4. Summary

Table 1 summarises several vascular flow ROM studies using SDR methods. We include the specific application, the reported accuracy compared to the FOM as a baseline, and the acceleration factor compared to the FOM. The accuracy reported for most ROMs was >90% and the acceleration factors ranged from 10^2^ to 10^5^. However, the ROMs are limited to investigating simple flow parameters, such as FFR, pressure drop or flow rates [60]. Gashi *et al.* [54] demonstrated that adding complexity (steady state to unsteady) reduces the acceleration offered by three orders of magnitude. Mirramezani & Shadden [59] presented a comprehensive study applying distributed 1D lumped parameter models to aortic, aorto-femoral, coronary, cerebrovascular, pulmonary and paediatric blood flow problems. Analytical expressions were used to allow the model to capture energy losses along vascular segments due to viscous dissipation, unsteadiness, flow separation, vessel curvature and vessel bifurcations.

**Table 1.** Various ROM papers using SDR for vascular flow problems. Acceleration is measured by comparing the time taken for one ROM evaluation with one FOM evaluation. This is the case for all tables presenting acceleration statistics, unless otherwise stated.

reference	method	application	accuracy	acceleration factor
Grinberg *et al.* [47]	1D	pulsatile intracranial blood flow	—	147 000^a^
Blanco *et al.* [55]	1D	FFR calculation in coronary arteries	98%	WCT: 302
				NT: 2870
Xiao *et al.* [56]	1D	baseline CCA	>99%	—
		baseline aorta	>98%	—
		aortic bifurcation	>98%	—
Papadakis & Raspaud [57]	1D (extended for stenosis)	wave propagation in stenotic vessels	>99%	—
Jonášová *et al.* [58]	1D	outlet flow rate in hepatic vein network	88%	—
			AW: 99%	—
Mirramezani & Shadden [59]	1D	flow rate and pressure calculations in various vascular domains	>93%	>1000
Gashi *et al.* [54]	2D steady	FFR calculation in coronary arteries	95%	162 000
	2D unsteady		98%	195

^a^Calculated by assuming a linear relationship between number of CPUs and simulation execution time.

Where accuracy is not reported, only qualitative ROM–FOM agreement was presented in the referenced paper.

AW, area-weighted; CCA, common carotid artery; FFR, fractional flow reserve; NT, normalised time (WCT × number of computation tasks); ROM, reduced order model; SDR, spatial dimension reduction; WCT, wall clock time.

#### 2.1.5. Conclusion

Zero-dimensional SDR models are suitable for global pressure/flow rate analysis of large regions of the cardiovascular system [45]. 1D models assume axisymmetric flow solutions to capture pressure and velocity pulse wave propagation [51]. 2D models can evaluate local flow fields with radial velocity variation in axisymmetric domains [61]. A prominent use of SDR models is providing boundary conditions to 3D models that incorporate information from significantly larger portions of the vasculature than it would be feasible to model in 3D [62–70]. In this way, SDR models can facilitate multi-scale spatial models that provide well-resolved 3D flow information in local regions of interest while still including the effect of distal or proximal regions. Zero-dimensional SDR models are unable to describe the nonlinearities that can arise in cardiovascular mechanics due to the convective acceleration term in the Navier–Stokes equations and/or the complex velocity–pressure relationship in distensible vessels [45]. 1D SDR models can approximate the effect of vessel wall elasticity on blood flow by adding a constitutive law that relates blood pressure to vessel cross-sectional area [51]. SDR models are generally only suitable for bulk velocity/pressure analysis in relatively simple geometries (i.e. axisymmetry is a valid assumption). They are typically unsuitable for complex multi-physics or multi-scale temporal problems but well suited for spatial multi-scale problems.

### 2.2. Proper orthogonal decomposition

SDR methods depend upon being able to apply geometrical simplifications (i.e. axisymmetry) or analogies with electrical circuit analysis to the vascular flow problem at hand to simplify the 3D Navier–Stokes equation into something easier and faster to solve. While SDR methods can be useful in capturing bulk quantities across large spatial scales, the applicability of these methods to other vascular flow complexities is limited. An alternative approach is to solve the expensive 3D Navier–Stokes equations and leverage the wealth of information contained in the data generated from these simulations to develop a ROM for the specific problem solved in the first instance. This is often referred to as a data-driven (or *a posteriori*) approach, as prior to ROM construction the FOM must be solved for some instances.

The method used to extract low-dimensional structures from high-dimensional data is key to any data-driven ROM. The most commonly used approach for this in fluid dynamics is the POD. POD was first introduced in fluid dynamics to analyse the structure of experimental turbulent flow and was later adopted for the purpose of efficient simulation and control of fluid flows [71,72]. POD extracts leading-order information from data in the form of orthogonal modes ordered by their energetic contribution to the data. In fluid flows, these modes typically capture spatial information contained within the data.

Before performing the POD, a snapshot matrix ***U*** is constructed by stacking columns of spatial data from different timesteps or input parameter configurations in a large matrix. A mean state derived by averaging over the timesteps or parameter configurations will often be subtracted from the snapshot matrix prior to performing the decomposition. Typically, the snapshot matrix will have many more rows than columns. POD is then performed by taking the singular value decomposition (SVD) of ***U***

2.4 $$U=\Phi \Sigma V^{*},$$

where ***Φ*** is a matrix of the left singular vectors, or POD modes, Σ is a diagonal matrix containing the singular values and V* is a matrix of right singular vectors. The success of POD in model order reduction stems from the observation that, in most complex physical systems, the meaningful behaviour of a system is captured by a low-dimensional subspace spanned by the first few POD modes. The singular values quantify the relative importance of each POD mode based upon its energetic contribution to the snapshot matrix. This knowledge makes it possible to truncate the system to a certain energy level by discarding the low-energy POD modes and retaining the high-energy modes.

POD-based ROMs have seen widespread application, including classical fluid dynamics problems [73–75], aerodynamics [76], FSI [77,78] and blood flow problems [30,78–83]. However, POD alone is not sufficient to build a ROM. POD provides a low-dimensional representation of the snapshots of the system, but the low-order representation must be combined with projection or interpolation techniques to build a ROM that can predict solution fields at new timesteps or input parameter configurations.

#### 2.2.1. Proper orthogonal decomposition with projection

Projection-based methods use the underlying governing equations of a system and POD modes to construct a ROM. The governing equations are projected onto the POD basis to derive a set of reduced equations embedded in this low-dimensional space. A common approach is to use the Galerkin projection (GP) [84,85]. POD-GP ROMs are among the most common ROMs that have been applied to vascular flow problems [30,82,83].

A POD-GP ROM can be derived by decomposing the velocity field ***u***(***x***, *t*)

2.5 $$u(x,t)\approx \sum_{\;j=1}^{N}a_{j}(t)\Phi_{j}(x),$$

where ***Φ***_*j*_ denote the POD modes and *a*_*j*_ are the temporal coefficients. The Galerkin projection of the Navier–Stokes equations is written as

2.6 $$\langle \Phi_{i},\frac{\partial u}{\partial t}+u\cdot \nabla u\rangle =-\langle \Phi_{i},\nabla p\rangle +\left\langle \Phi_{i},\frac{1}{\text{Re}}\nabla^{2}u\right\rangle ,$$

where 〈 · , · 〉 represents the inner product. Following some algebraic manipulation using the decomposition from equation (2.5), the POD-GP ROM can be written as [86]

2.7 $$\begin{array}{r} \frac{\text{d}a_{i}(t)}{\text{d}t}=A_{i}+\sum_{\;j=1}^{N}B_{ij}a_{j}(t)+\sum_{\;j=1}^{N}\sum_{k=1}^{N}C_{ijk}a_{j}(t)a_{k}(t),\;i=1,\ldots , \end{array}N.$$

*A*_*i*_, *B*_*ij*_ and *C*_*ijk*_ are tensors determined by the specific form of the governing system. The functional forms of the coefficient tensors are

2.8 $$\begin{array}{rl} & A_{i}=-\frac{1}{\text{Re}}\langle \nabla \Phi_{i},\nabla \bar{u}\rangle -\langle \Phi_{i},(\bar{u}\cdot \nabla )\bar{u}\rangle \\ & B_{ij}=-\langle \Phi_{i},(\bar{u}\cdot \nabla )\Phi_{j}\rangle =\langle \Phi_{i},(\Phi_{j}\cdot \nabla )\bar{u}-\frac{1}{\text{Re}}\langle \nabla \Phi_{i},\nabla \Phi_{j}\rangle \\ \mathrm{and}\; & C_{ijk}=-\langle \Phi_{i},(\Phi_{j}\cdot \nabla )\Phi_{k}\rangle , \end{array}\}$$

where $\bar{u}=\int_{0}^{T}u(x,t)\;dt$ is the time-averaged flow [29]. The double sum in equation (2.7) arises due to the nonlinearity of the Navier–Stokes equations and is responsible for the slower ROM speeds and greater storage demands required in the case of nonlinear systems.

***Nonlinearity*** When applied to problems governed by nonlinear equations, POD-GP does not fully decouple the ROM equations from the FOM, as the algebraic form of the ROM equations retains dependence on the FOM [29]. This means that the algebraic operators for the ROM need to be recomputed at every iteration of the system, which limits the acceleration that this approach can offer for the target application of vascular flow. It is possible to overcome this issue by using hyper-reduction techniques, such as the discrete empirical interpolation method (DEIM), which approximates the algebraic operators instead of calculating them exactly [87]. Buoso *et al.* [30] employed this technique in a POD-GP-DEIM ROM to evaluate coronary blood flow, and found an acceleration by a factor of 25 for this method compared to the FOM.

***Geometric complexity*** Complex geometric variability can be modelled by POD-Projection methods, as the POD modes can be made to contain spatial information about the geometry used to generate the data by mapping them back to a fixed reference geometry. However, applying any kind of ROM to a geometry not included in the training data is typically very challenging. In particular, when looking at vascular flow, the variability in morphology from one person to the next can be extreme, with entire vascular segments sometimes missing in certain regions [88]. In some cases, such as when modelling relatively simple features such as stenosis in reasonably straight vessels, it is possible to parameterise the geometric variation and include these parameters as input to the ROM, as in [30]. However, for pathologies such as intracranial aneurysms, where blood flow is highly dependent on the morphology, the number of parameters needed and the amount of high-fidelity data required can be prohibitive. Buoso *et al.* [30] demonstrated the use of DEIM to accelerate mesh generation by a factor of 10, which could help improve the overall efficiency of a simulation pipeline studying blood flow in multiple geometries.

***Multi-physics*** Provided the governing equations are known and data can be generated for the system, POD-Projection techniques are suitable for multi-physics problems. A common multi-physics application of POD-Projection is to FSI problems [89–91]. Ballarin & Rozza [92] applied a POD-GP ROM to three idealised 2D FSI problems, including a parameterised valve configuration. The ROM showed good qualitative agreement across all cases and an acceleration factor of the order of ten.

***Multi-scale (time)*** While POD-Projection ROMs are able to reduce simulation times significantly, the long-term stability of the ROM for unsteady flow problems is not guaranteed [29,93]. This instability can be related to the truncation of the POD basis, the violation of boundary conditions, or an inherent lack of numerical stability [86]. Various stabilisation techniques can overcome these issues, such as balanced truncation and balanced POD [29], pressure stabilisation [74], or adding corrective terms to the ROM equations to increase dissipation [94]. Adding these stabilisation techniques to a ROM may increase its long-time accuracy, but will likely come at the cost of increased computational demands [90]. Lassila *et al.* [29] noted that periodically driven inflow problems have been shown to demonstrate accurate long-term predictions. Given the quasi-periodic nature of vascular flow, this may imply that ROM stability is satisfactory in this context. However, care must be taken to train the ROM with data that are representative of the entire cardiac cycle. Flow features will exhibit strong time dependence due to the pulsatile nature of vascular flow [14]. As a result, training a ROM using data from only one part of the cardiac cycle (e.g. flow acceleration) is unlikely to produce a ROM capable of accurately predicting the flow at another time (e.g. diastole).

***Multi-scale (space)*** The spatial information is contained within the POD modes when constructing a POD-projection ROM. The number of spatial degrees of freedom is the same as the number of rows in each POD mode, which means that data and computing requirements for POD ROMs will increase as the mesh size grows. Furthermore, as POD requires input data from a FOM, using a refined mesh that captures fine flow details could lead to prohibitive run times when solving the FOM. This means that POD-Projection ROMs are often unsuitable for problems where large regions of the vasculature need to be modelled. A possible strategy to mitigate this issue is to couple a POD-Projection ROM with boundary conditions that are derived from a SDR ROM. Using this technique allows for the high spatial resolution of the POD-Projection approach in the region of interest while still accounting for the effects of the proximal and/or distal vasculature using the SDR model. This technique has been used in various haemodynamics studies to couple high-fidelity 3D models to SDR models, but POD-Projection ROMs have not been used for the 3D model [67,81,95].

***Other comments*** While using the underlying governing equations is thought to improve the robustness of projection-based ROMs, it is also a weakness regarding the ease of implementation. Constructing a projection-based ROM requires explicit use of the underlying numerical implementation of the FOM, which may not be available or straightforward to use. In particular, when solving fluid dynamics problems, researchers often turn to commercial software for which source code is not readily available. This can hinder incorporating projection-based ROMs into simulation pipelines that are not built upon open-source software. Equation-free or non-intrusive methods offer an alternative strategy that mitigates these issues.

#### 2.2.2. Proper orthogonal decomposition with interpolation

An alternative to projection-based ROMs is to use interpolation-based methods. Given a snapshot matrix ***U***, with SVD given by $U=\Phi \Sigma V^{*}$, it is possible to reconstruct each column of ***U*** using

2.9 $$u^{n}(x,t;\mu )=\sum_{\;j=1}^{N}a_{j}^{n}(t;\mu )\Phi_{j}(x),$$

where ***μ*** are the parameter configurations contained in the snapshots, $a_{j}^{n}(t;\mu )$ are a set of time and parameter dependent coefficients, *N* is the number of truncated POD modes retained for the ROM and **Φ**_*j*_ are the POD modes. ***a***^*n*^ are a set of temporal coefficients that can be considered as a path through the coordinate system given by **Φ** [76]. The goal of POD-Interpolation is to predict the trajectory of the system under a new set of parameter values by using interpolation between the trajectories of previously computed parameter values. To perform the interpolation step, authors have turned to various techniques, such as linear interpolation [96], radial basis functions (RBFs) [76,97,98], Taylor series methods, or Smolyak grids [99]. Once calculated, the new set of coefficients can be multiplied by the retained POD modes to efficiently calculate the solution field of interest for a new parameter configuration or time point.

***Nonlinearity*** POD-Interpolation is a non-intrusive method, meaning that no modification of the underlying FOM numerical code is required. This means that the ROM is agnostic to the system it is being applied to and, therefore, POD-Interpolation does not suffer the same drawbacks as POD-Projection when applied to nonlinear systems. This does not guarantee that results with a POD-interpolation approach will be accurate for a nonlinear system, but the speed of the model is not drastically reduced in this scenario as can be the case when using POD-Projection on nonlinear problems.

***Geometric complexity*** Similarly to POD-Projection methods, POD-Interpolation is suitable for complex-shaped individual geometries due to the POD modes containing rich spatial information. However, the success of this approach is also limited when applied to geometries that were not included in the training data. Girfoglio *et al.* [69] applied POD-Interpolation methods to patient-specific aortic blood flow in the presence of a left ventricular assist device, but only constructed their ROM for a single patient geometry. Geometric parameterisation approaches have been applied to POD-Interpolation methods, but not in the context of vascular flow problems [76].

POD-Interpolation approaches can be applied to sub-domains of the FOM domain used to generate the snapshots. For example, if high-fidelity data were generated for a vessel with an aneurysm, it is possible to build a POD-Interpolation ROM only for the aneurysm rather than the full geometry. This can further accelerate the ROM, as the number of data and interpolation operations required is reduced. This feature of POD-Interpolation ROMs gives them an advantage when modelling flow in complex geometries where dense volumetric meshes are required (e.g. when modelling a flow-diverting stent), as the amount of data is vastly reduced without affecting the model performance.

***Multi-physics*** POD-Interpolation techniques have been applied infrequently to multi-physics problems. Xiao *et al.* [77] used a non-intrusive POD-RBF ROM for one-way and two-way coupled FSI problems and found acceleration factors of order 10^5^–10^6^ while showing qualitative ROM-FOM agreement. Hajisharifi *et al.* [98] applied a POD-RBF ROM to a fluidised bed problem. Compared to the FOM, the POD-RBF ROM provided an acceleration factor of order 10^5^ and an accuracy of approximately 99% when reconstructing the time evolution of the Eulerian and Lagrangian fields. They tested local and global POD approaches and found the local calculation of POD bases produced a more accurate and efficient ROM.

***Multi-scale (time)*** Similarly to POD-Projection techniques, POD-Interpolation methods do not have any guarantee of long-term solution stability.

***Multi-scale (space)*** In principle, POD-Interpolation ROMs can be coupled with 0D/1D models for boundary conditions by including the coupling parameters describing the inflow/outflow conditions in the ROM construction. When evaluating the POD-Interpolation ROM, one can obtain the boundary condition parameter input from the output of the 0D/1D boundary condition model and use this to evaluate the 3D flow field using the ROM. In this way, POD-Interpolation approaches can be suitable for modelling highly resolved regions of interest in 3D while conscribing to the effects of the peripheral vasculature. This POD-Interpolation-SDR approach is yet to be applied to vascular flow, but coupling 0D/1D models with 3D computational fluid dynamics (CFD) is common [67,81,95].

***Other comments*** Walton *et al.* [76] noted that POD-Interpolation, when all POD modes are retained, is equivalent to performing element-wise interpolation across all spatio-temporal coordinates. Therefore, the maximum accuracy for a POD-Interpolation ROM will be bounded by the element-wise interpolation error. For this reason, the acceleration offered by POD-Interpolation ROMs should not only be calculated relative to the high-fidelity CFD model, but also relative to the cost of performing element-wise interpolation of the solution field. Despite this limitation, relative to element-wise interpolation, POD-Interpolation is still capable of vastly reducing the number of interpolation operations required to calculate a new solution and the amount of data that needs to be stored offline.

***Summary*** POD-Projection and POD-Interpolation techniques have been applied to a wide range of vascular flow problems, including blood flow in tetralogy of Fallot patients [80,81], coronary blood flow [30,82,100], aneurysm blood flow [101], aortic blood flow [69,102] and FSI problems [92]. Tables 2 and 3 demonstrate that POD-Interpolation ROM techniques typically accelerate by factors ranging from 10^2^ to 10^6^, while acceleration factors for POD-Projection ROMs range from 10^1^ to 10^3^. Wang *et al.* [96] compared POD-GP and POD-Interpolation approaches for steady-state heat conduction problems with different numbers of parameters. They found that the POD-GP approach was more reliable, with better performance as the number of parameters grew. POD-Interpolation may require more snapshots than POD-GP to achieve similar accuracy, so despite the faster evaluation times of POD-Interpolation, the overall offline cost to build a ROM of equal accuracy to the POD-GP ROM may be greater. Xiao *et al.* [99] and Xiao *et al.* [97] performed two studies comparing POD-GP with various POD-Interpolation techniques (Taylors, Smolyak, RBF interpolation). In both studies, the interpolation-based ROMs were found to be approximately one order of magnitude faster while maintaining good accuracy relative to the high-fidelity model.

**Table 2.** Various ROM papers using POD for vascular flow and other selected problems.

reference	method	application	accuracy	acceleration factor
*general applications*				
Xiao *et al.* [77]	POD-Interpolation (RBF)	one-way FSI: flow past a cylinder	—	727 000
		two-way FSI: free-falling square	—	73 200
		FSI: bending beam	—	257 000
Hajisharifi *et al.* [98]	local POD-Interpolation (RBF)	fluidised bed time evolution	Eulerian field 98.9%, Lagrangian field 98.4%	200 000
		parametric fluidised bed	88.8%	
*vascular flow applications*				
McLeod *et al.* [80]	atlas-based POD	ToF PA flow: case 1	$∼70{\%}^{a}$	—
		ToF PA flow: case 2	$∼80{\%}^{a}$	—
		ToF PA flow: case 3	$∼50{\%}^{a}$	—
		ToF PA flow: case 4	$∼80{\%}^{a}$	—
Guibert *et al.* [81]	atlas-based POD	ToF PA flow: patient 7	Δ*p* 95.7%, outlet flow 96.0%	∼1.33
		ToF PA flow: patient 13	Δ*p* 93.9%, outlet flow 97.7%	
Buoso *et al.* [30]	POD-GP-DEIM	FFR calculation in coronary stenosis: case 1	FFR 98%, min. *p* accuracy 70%	25
		FFR calculation in coronary stenosis: case 2	FFR 92%, min. *p* accuracy 90%	
Ballarin & Rozza [92]	POD-GP	fluid problem on moving domain	—	30
		stationary FSI of parameterised idealised valve	—	16
		unsteady FSI of parameterised channel	—	10
Ballarin *et al.* [82]	POD-GP	coronary blood flow with varying physical and geometric parameters	>99%	100
Ballarin *et al.* [100]	POD-GP	coronary blood flow with varying physical and geometric parameters	>99%	1530^b^; 100^b^
Han *et al.* [101]	POD-GP	aneurysm blood flow with varying PI	>95%	2410
Zainib *et al.* [103]	POD-GP	coronary artery bypass grafts	>99%	9^c^
Girfoglio *et al.* [102]	POD-Interpolation (RBF)	aortic flow with LVAD	*p* 99.5%, WSS 92.3%, *u*_*x*_ 91.5%, *u*_*y*_ 87.8%, *u*_*z*_ 88.6%	240
Girfoglio *et al.* [69]	POD-Interpolation (RBF)	aortic flow with LVAD: case 1 (PF 3.45 l min^−1^)	*p* 99.8%, WSS 95.9%, *u*_*x*_ 95.0%, *u*_*y*_ 92.2%, *u*_*z*_ 94.2%	7 200 000
		aortic flow with LVAD: case 2 (PF 4.35 l min^−1^)	*p* 99.5%, WSS 92.8%, *u*_*x*_ 90.3%, *u*_*y*_ 86.5%, *u*_*z*_ 90.7%	

^a^Maximum error estimated from graph in paper and used to calculate minimum accuracy (which occurs close to systole).

^b^Authors report computational savings of 99% (therefore acceleration factor of 100). In total, 1530 acceleration factor is calculated from simulation times presented for ten patients in table 2 of [100].

^c^Mean acceleration calculated across three test cases in table 1 of [103].

DEIM, discrete empirical interpolation method; FFR, fractional flow reserve; FSI, fluid–structure interaction; GP, Galerkin projection; LVAD, left ventricular assist device; *p*, pressure; Δ*p*, pressure drop; PA, pulmonary artery; PI, pulsatility index; POD, proper orthogonal decomposition; RBF, radial basis functions; ROM, reduced order model; ToF, tetralogy of Fallot; *u*_*x*_, x-component of velocity; WSS, wall shear stress.

**Table 3.** ROM papers comparing POD-Projection and POD-Interpolation approaches for various applications.

reference	method	application	accuracy	acceleration factor
Xiao *et al.* [99]	POD-GP	flow past a cylinder	—	10
	POD-I (Taylors)		—	260
	POD-I (Smolyak)		—	390
Xiao *et al.* [97]	POD-GP	lock exchange	—	12
		flow past a cylinder	—	10
	POD-I (RBF)	lock exchange	—	496
		flow past a cylinder	—	779
Wang *et al.* [96]	POD-GP	four-variable heat conduction	99.81%	—
		six-variable heat conduction	98.17%	—
	POD-I	four-variable heat conduction	>99.99%	—
		six-variable heat conduction	∼50%	—

GP, Galerkin projection; POD, proper orthogonal decomposition; POD-I, POD-Interpolation; RBF, radial basis functions; ROM, reduced order model.

***Conclusion*** POD-Projection and POD-Interpolation approaches have been applied to nonlinear, geometrically complex, multi-physics vascular flow problems. Both of these approaches can be coupled to 0D/1D models to capture multi-scale phenomena across large spatial scales in the vasculature. Geometric parameterisations can be incorporated into POD-based ROMs in an attempt to build models suitable for unseen geometries, but these models are limited in their generality and in the complexity of geometry they can model with a reasonable number of parameters. Attempts to build POD-based ROMs that are entirely general to geometry have seen either large errors [80] or minimal acceleration [81]. POD-based ROMs are often unsuitable for problems with large time scales, as the long-term stability of the POD modes is not guaranteed.

### 2.3. Dynamic mode decomposition

Dynamic mode decomposition (DMD) was originally developed by Schmid [104] for analysing spatio-temporal data from simulations and experiments. Modes are extracted from the data and can then be used to describe the physical mechanisms present in the data or for dimensionality reduction. For ROM construction, DMD can provide an alternative technique to POD for extracting leading-order modes from data. DMD trades the optimal reconstruction property of POD for physical interpretability, as the eigenvalue associated with each mode provides quantitative information on the oscillation frequency or growth/decay rate of the given mode [105].

Both DMD and POD use the SVD, but the difference arises in the construction of the snapshot matrix prior to performing SVD. In POD, the snapshot matrix is given by ***U*** = [***u***_1_, … , ***u***_*N*_]. For DMD, the snapshot matrix is first divided into two submatrices, ***U***_1_ = [***u***_1_, … , ***u***_*N*−1_] and ***U***_2_ = [***u***_2_, … , ***u***_*N*_]. The goal of DMD is to compute an approximation to the matrix ***A***, where ***U***_2_ ≈ ***AU***_1_ [106]. To do this, SVD is applied to ***U***_1_ and the resulting decomposition is used to calculate the pseudoinverse of ***U***_1_, which is then used to calculate ***A***. Thus, DMD finds a best-fit linear model that approximates the underlying time dynamics present in the data. In DMD, *N* will typically be a set of timesteps for the evolution of the system for one set of parameter values. Using the DMD model, an initial state can be propagated forward in time at a low cost. DMD ROMs are non-intrusive by being equation-free and entirely data-driven.

Since its inception, numerous extensions to DMD have been proposed to help tackle complexities such as nonlinearity, varying characteristic time scales in a given application, or handling externally driven data sequences. These extensions are thoroughly presented in [33]. Despite its growing use as a tool for analysing complex spatio-temporal data, DMD has seen limited application to vascular flow. We will discuss the applicability of DMD and its extensions to modelling vascular flow.

#### 2.3.1. Nonlinearity

DMD aims to find an optimal linear model based on data. The underlying system in blood flow problems is nonlinear but the strength of this nonlinearity will vary depending upon the application. Habibi *et al.* [105] found that more DMD modes are required in an aneurysm model than in a stenosis model to achieve a particular reconstruction accuracy, highlighting the problem-specific nature of the complexity of vascular flow. In cases where nonlinearity is strong, a large number of measurements of the field of interest may be required to ensure the nonlinearity is captured in the reduced model. Extended DMD (EDMD) is an approach designed to help with this issue by using nonlinear functions of the measurements as input to the DMD algorithm [33,107].

#### 2.3.2. Geometric complexity

Similarly to POD modes, DMD modes contain spatial information, so this approach is well suited to constructing ROMs for individual complex geometries. Habibi *et al.* [105,108,109] have demonstrated the use of DMD to identify blood flow structures in cerebral aneurysms and stenosis models. However, as with POD, using DMD to evaluate flow fields in an unseen geometry is very challenging. DMD is a less well-established technique than POD, so few (if any) attempts have been made to tackle this problem.

#### 2.3.3. Multi-physics

DMD is suitable for multi-physics problems as the decomposition can be applied separately to each field. DMD can also be used to identify spectral coherence between each field in multi-physics applications, which can help to improve understanding of the problem. So far, the main use of DMD in multi-physics problems is to study FSI. Rodríguez-López *et al.* [110] used DMD to capture spatio-temporal evolution of flow over a flexible membrane wing using experimental data. They found that basic DMD could not reconstruct the fields accurately. Instead, they used high-order DMD (DMDho), developed by Le Clainche & Vega [111]. Where basic DMD only uses the previous snapshot, DMDho estimates each snapshot as a linear combination of a number of previous snapshots, thus improving performance in regimes where the FSI was stronger. This suggests that as the complexity of the system increases, accurate propagation of the time dynamics may require more than just the previous snapshot. This is worth considering when adding complexity (e.g. vessel elasticity, thrombosis models, device interactions) to vascular flow DMD models.

#### 2.3.4. Multi-scale (time)

DMD ROMs are perhaps most beneficial for problems of complex temporal nature. A DMD ROM is inherently designed to uncover time dynamics in a system and then propagate the reduced system forwards in time. Vascular flow is often modelled as periodic, with results from a single cardiac cycle taken to be representative of the flow for all time. This assumption can break down when autoregulation occurs or when complex long-term physiological phenomena, such as blood clotting, occur. The period of a cardiac cycle is roughly one second, whereas processes such as blood clotting can occur over a period of months. Multi-resolution DMD (DMDmr) provides a way to robustly separate complex systems into a hierarchy of multi-resolution time components [112]. DMDmr uses iteratively shorter snapshot sampling windows and recursive extraction of DMD modes from slow to fast time scales, which improves the predictions for short-time future states. This technique has been further generalised by Dylewsky *et al.* [113]. Provided with the appropriate data, DMDmr may be able to produce ROMs that can capture both long- and short-term effects of blood flow. Identifying a ROM for long-term effects (clotting, plaque build-up etc.) may be particularly useful in reducing the cost of vascular models, as current approaches are too expensive to simulate these processes for the time scales over which they occur [3]. Another approach to handle complex temporal patterns is multi-stage DMD (mDMD) [105]. mDMD divides a temporal system into stages and applies DMD to each stage in turn. This allows more DMD modes to be used during periods with a more complex flow, while reducing the number of modes required when the flow is simpler, as demonstrated by Habibi *et al.* [105]. This approach can improve the efficiency of the ROM and reduce data storage requirements, but does not extend the original DMD method to more complex problems.

#### 2.3.5. Multi-scale (space)

DMD modes are local to wherever the high-fidelity data were generated, so using this approach for large regions of the vasculature is not possible without generating enormous amounts of high-fidelity data. However, DMD with control (DMDc) allows for input controllers to be integrated into the DMD algorithm. Habibi *et al.* [105] used inlet velocity as a controller for cardiovascular flow. It may be possible to extend this approach to account for other flow parameters or boundary conditions, thus allowing the inexpensive DMD ROM to be coupled to 0D/1D SDR models that account for the large-scale flow changes in the vasculature.

#### 2.3.6. Summary

Despite DMD being used as a ROM technique, very few papers directly compare the efficiency of the DMD ROM with the FOM used to generate the training data. Table 4 highlights a few studies that did evaluate the DMD ROM efficiency. From this, we can see speed-ups ranging from ∼10^0^ to 10^2^. This acceleration seems small, but given the non-iterative equation-free nature of DMD ROMs, it is likely that they can provide more acceleration than this in some scenarios. Furthermore, Lu & Tartakovsky [115] included offline calculation times when determining the ROM speed-up, so higher acceleration values would be found if they only compared the online evaluation time with the FOM.

**Table 4.** ROM papers using DMD for various applications.

reference	method	application	accuracy	acceleration factor
*general applications*				
Bourantas *et al.* [114]	DMD	tumour ablation treatment simulation	>99.8%	∼13–37
Lu & Tartakovsky [115]	Lagrangian DMD	1D advection	—	0.21^a^
		1D advection–diffusion	—	581^a^
		1D inviscid Burgers equation	—	0.81^a^
		1D viscous Burgers equation	—	993^a^
	POD-GP	1D advection	—	0.15^a^
		1D advection–diffusion	—	84.2^a^
		1D inviscid Burgers equation	—	0.09^a^
		1D viscous Burgers equation	—	69.4^a^
Beltrán *et al.* [116]	DMDho-augmented FOM	1D Ginzburg–Landau equation	—	6–254^b^

^a^Authors include offline calculation times in DMD computational time, hence the ROM sometimes being slower than the FOM [115].

^b^Authors define speed-up as ratio of total simulation time to the sum of the time-lengths of the snapshot computational intervals, which is a particular definition suitable for their method [116].

DMD, dynamic mode decomposition; DMDho, high-order DMD; GP, Galerkin projection; FOM, full-order model; ROM, reduced order model; POD, proper orthogonal decomposition.

Only a few papers in the literature use DMD for vascular flow problems. Habibi *et al.* [105] used multi-stage DMD with control (mDMDc) to reveal hidden low-dimensionality in patient-specific blood flow in coronary stenosis and cerebral aneurysms. They found that mDMDc requires fewer modes than DMD to reconstruct the velocity fields to a given accuracy, but these modes were not used to construct a ROM. Habibi *et al.* [109] used DMD for data assimilation in Womersley flow, 2D idealised aneurysm flow and 3D real aneurysm flow, but in this instance the DMD analysis was not used to construct a ROM. Di Labbio & Kadem [117] performed POD and DMD analysis of left ventricular flow and found that while DMD requires more modes to achieve a particular energy level, it also preserves global particle advection using fewer modes. Another important point to consider when using DMD for vascular flow is that due to the periodic nature of the flow, unstable modes will either decay or grow over time, thus potentially under- and over-influencing the dynamics as time goes on [117].

#### 2.3.7. Conclusion

DMD can be used to construct reduced order linear dynamical systems from data that approximate underlying nonlinear dynamics. DMD ROMs can be inexpensively propagated forwards in time or used to extract coherent structures from data. DMD offers the benefit of having an associated frequency attached to each mode, thus providing interpretability (i.e. growth/decay/oscillation for each mode). DMD modes contain spatial information so this approach can be used to model individual complex geometries. DMD models are typically built with time as the only input parameter, so parametric DMD ROMs are rare; however, very recent work has begun to investigate this by adding interpolation into the DMD approach [118]. DMDc offers the potential to include input controllers into a DMD model, so this approach can be used to include the effects of, for example, varying inlet flow rate [105]. The input controllers could also potentially be boundary conditions derived from 0D/1D blood flow models, thus allowing DMD ROMs to account for larger portions of the vasculature. DMD can be applied to multi-physics problems; however, a high-order DMD approach may be required to correctly reconstruct the fields of interest [111]. DMD ROMs are not commonly applied to vascular flow problems to date. A promising application of DMD in vascular flow is to problems where evaluating the long-term effects is not possible with conventional models. For these problems, DMD could perhaps be used to construct an efficient ROM for the time dynamics of long-term blood flow phenomena.

### 2.4. Other techniques

There are various other ROM techniques that have not been as widely used as those discussed previously. Herein, we will discuss two of those techniques, the reduced basis (RB) method, which has seen some application to vascular flow problems, and the proper generalised decomposition (PGD), which has not been applied to vascular flow modelling.

#### 2.4.1. Reduced basis

The RB method is usually applied to the fast solution of parameter-dependent problems [29,119,120]. Similarly to POD-based ROMs, the RB method uses a set of snapshots of the FOM. Whereas POD uses the SVD to extract an optimal basis from the snapshots, the RB method is more general and can use various alternative approaches (e.g. Gram–Schmidt orthonormalisation [121]) to construct a basis spanning a sub-space of typically much lower dimension than that of the full-order solution manifold. RB methods often employ a greedy procedure for basis construction, whereby optimal snapshots are computed based upon an *a posteriori* error estimation [122]. A key advantage of the greedy approach is that the specific dynamics of the problem at hand guide the sample selection process [26]. Following basis construction, a Galerkin projection is often applied to build the ROM, similarly to POD-Projection ROMs.

The RB method has seen some application to vascular flow problems. Manzoni *et al.* [123] used this approach with RBF for interpolating the geometric parameters to calculate flow fields in 2D parameterised carotid artery bifurcation geometries. For two test cases of global deformations of the carotid branches and stenosis near the carotid sinus, they achieve speed-ups of 96 and 88 times, respectively. Lassila *et al.* [124] applied the RB method to inverse problems in flow through stenosed arteries and in optimal shape design for femoropopliteal bypass grafts, reporting estimated speed-ups of 30–175 times. While effective in predicting downstream shear rates in the stenosis problem and in identifying optimal design configurations, the models were only applied to 2D steady-state problems. Colciago & Deparis [125] combined POD and the RB method, specifically the greedy algorithm, to build a ROM for a haemodynamics problem, noting CPU time gains of order 10^3^. The application was to a femoropopliteal bypass problem, which was modelled using a 3D reduced FSI formulation, highlighting the suitability of the RB approach to multi-physics applications. The authors note that the greedy enrichment scheme can favour reducing the error in certain variables, especially when the quantities in the problem are of different orders of magnitude, so care should be taken in building an appropriate error estimator for multi-physics applications. Aside from vascular flow applications, the RB method has been applied to various other nonlinear Navier–Stokes problems [126,127], including FSI problems [128]. Coupling the parametric RB method to boundary conditions derived from 0D vascular models is possible in order to capture some multi-scale spatial effects.

#### 2.4.2. Proper generalised decomposition

PGD generalises POD using separated representations while avoiding the need for any *a priori* knowledge about the solution [129]. Not using snapshot generation allows PGD to be applied to previously unsolved problems, which POD, DMD and RB ROMs are mostly incapable of. For a problem defined in space of dimension *D*, PGD provides an approximate solution *u*^*N*^ in the separated form

2.10 $$u^{N}(x_{1},\ldots ,x_{D})=\sum_{i=1}^{N}F_{i}^{1}(x_{1})\times \cdot \cdot \cdot \times F_{i}^{D}(x_{D}).$$

The PGD approximation is a sum of *N* functional products involving *D* functions $F_{i}^{j}(x_{j})$ [130]. PGD solutions are constructed by successive enrichment, where a functional product *F*_*n*_ is determined using the functions from the previous *n* − 1 steps. It should be noted that each enrichment step involves solving a nonlinear problem by means of a suitable iterative process. In PGD, both the number of terms *N* and the functions *F* are unknown *a priori*, making PGD an *a priori* ROM method. In a typical separation of variables, the coordinates *x*_*i*_ could be space and time coordinates, but in PGD additional coordinates can be included for problem-specific inputs such as boundary conditions or material parameters. Furthermore, if *M* nodes are used to discretise each of the coordinate spaces, the total number of PGD unknowns is *N* × *M* × *D* instead of the *M*^*D*^ degrees of freedom found in standard mesh-based discretisations [130]. When the solution field is sufficiently regular, the number of terms *N* will be relatively small, highlighting how PGD overcomes the curse of dimensionality [131].

PGD was initially developed for solving time-dependent nonlinear problems in structural mechanics [132]. It has since been applied to rheology [133] and the incompressible Navier–Stokes equations [131]. Chinesta *et al.* [133] noted a speed-up of the order of 10^2^ when using PGD for a transient rheology problem. Dumon *et al.* [131] found a speed-up of approximately 100 times for a 2D stationary diffusion problem, whereas a speed-up of 5–10 times was found for various Navier–Stokes problems, the most complex of which was a 2D lid-driven cavity flow. PGD has also been applied to multi-scale in time applications, where it is possible to separate the time dimension (1D in nature) into a multi-dimensional time space; however, in this study the authors are not able to draw conclusions on the efficiency of the ROM [134]. PGD has also seen application to multi-scale in space and multi-physics problems, where the authors highlight that the savings due to PGD increase with problem complexity [135,136]. Despite its potential usefulness in complex problems with known/unknown equations, PGD has not seen as widespread use as other reduced order techniques.

## 3. Accelerating simulations with machine learning

**Figure 3.**
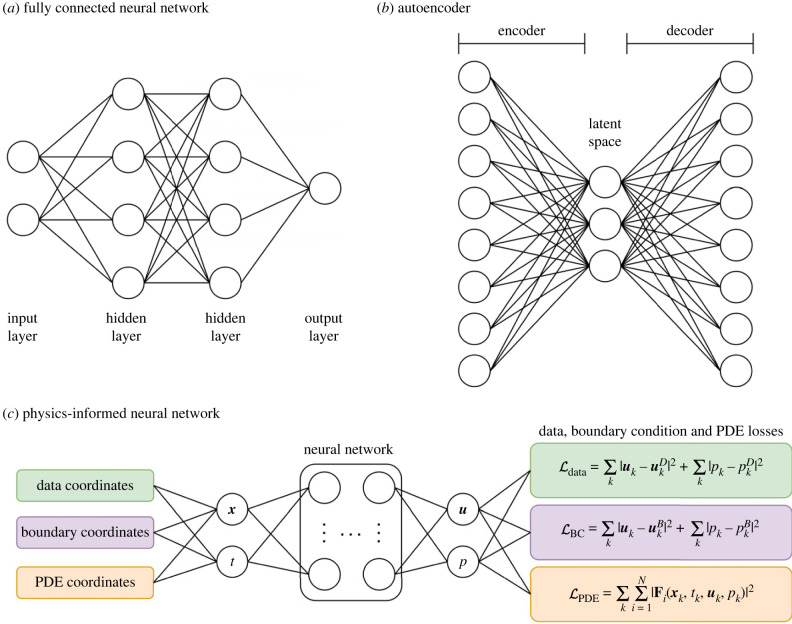
Selected neural network designs that can be used for simulation acceleration. (*a*) A fully connected neural network with two inputs, two hidden layers with four neurons per layer and one output. (*b*) A fully connected autoencoder, consisting of an encoder, a latent space and a decoder. (*c*) A physics-informed neural network, where physical constraints based on partial differential equations (PDEs) and boundary conditions (BCs) are included in the loss function of the network. ***x*** is position, *t* is time, ***u*** is velocity, *p* is pressure, superscript *D* or *B* means data or boundary point, **F**_*i*_ are *N* residual equations.

Machine learning is a branch of artificial intelligence that excels at extracting underlying patterns in data. The basic building block of many machine learning algorithms is the neural network, shown in figure 3. Neural networks consist of a collection of processing units, called neurons, and a set of directed weighted synaptic connections between the neurons. The connections between neurons symbolise the passing of information between neurons, with a fully connected neural network (FCNN) meaning that all neurons in a given layer receive information from all neurons in the previous layer and pass information to all neurons in the subsequent layer. Each neuron processes the information it receives via some calculations and produces an output. The final layer is referred to as the output layer, where the final output of the network is produced. The fully connected neural network in figure 3 has two inputs, two hidden layers with four neurons per layer and one output. The objective of the network is to approximate a mapping between the input and output variables, given data to learn from. In vascular flow modelling, the inputs may be variables like space, time or Reynolds number and the outputs may be velocity, pressure or other variables of interest.

Each neuron is characterised by three functions: the propagation function, the activation function and the output function. The propagation function converts the vectorial input from the previous layer’s outputs into a scalar input. The activation function quantifies the extent to which a particular neuron is active by applying a chosen function to the net input, such as the hyperbolic tangent or rectified linear unit functions [137]. Including activation functions for several sequential layers allows the deep network to approximate nonlinear mappings from inputs to outputs. The output function calculates the scalar output of a neuron based upon its activation state. Each neuron has a trainable weight associated with it, and each layer often has a trainable bias. These weights and biases are the network parameters that are optimised through training.

For a supervised learning problem, training data consist of a set of inputs with known outputs. During the training procedure, input data are passed through the network to give an output that is compared to the ground truth values for the output. A loss function is used to quantify the discrepancy between the network output and the ground truth output. The parameters associated with the network are optimised, typically through back-propagation and gradient descent algorithms, in order to minimise the loss [138]. Once the network has been trained to accurately match predictions for the training dataset, it can be used for input data where ground truth output values are unknown. Typically, the accuracy of the network will be assessed by evaluating its output on a dataset that was not used in training, or through procedures such as cross-validation. A trained neural network can be considered to approximate a function that maps the input data to the output data. Hornik *et al.* [139] have demonstrated the approximation power of sufficiently large and deep networks. Variations on basic neural networks include autoencoders, convolutional neural networks (CNNs), recurrent neural networks (RNNs) and physics-informed neural networks (PINNs), among others [140].

Machine learning and deep learning have both employed neural networks to great effect in various classification and regression tasks in fields such as computer vision and natural language processing [141,142]. Common across all learning-based strategies is the utilisation of data and the framework of an expensive up-front training stage preceding a cheap inference stage when evaluating the model for new data. In this way, machine learning approaches bear resemblance to ROM methods. A benefit of machine learning compared to ROMs is that the operations used in machine learning are highly parallelisable, which allows them to be trained and tested using highly parallel computing hardware, such as graphics processing units (GPUs). This can reduce the time taken for training and inference, which is driving the growing interest in using machine learning-based simulation methods for acceleration.

Machine learning can be used in conjunction with ROMs, where the dimensionality reduction inherent to the ROM provides acceleration and machine learning is used to improve or replace some aspect of the ROM. For example, when constructing an interpolative ROM, such as in the POD-Interpolation method, using a neural network for interpolation can produce a ROM capable of outperforming POD-GP ROMs in terms of both acceleration and accuracy for certain applications [143–145]. Alternatively, machine learning can be used in place of conventional simulation methods to directly infer solution fields or other quantities of interest from inputs such as medical images and point clouds of spatio-temporal coordinates [36,37]. In this instance, the machine learning model itself provides acceleration relative to the FOM, either through reduction of the dimensionality of the problem or through exploitation of parallel computing hardware.

### 3.1. Machine learning reduced order models

#### 3.1.1. Machine learning-augmented reduced order models

Various attempts have been made to augment ROMs with machine learning. Neural networks (NNs) are adept at interpolation, so using them in POD-Interpolation ROMs is a natural choice. Hesthaven & Ubbiali [143] were among the first to apply a POD-NN ROM to parameterised steady-state PDEs (the Poisson equation and lid-driven cavity problems). In this model, the network approximates a mapping from the input parameter vector (including, e.g. material/geometry parameters) to the ROM coefficients. The POD-NN approach offers similar accuracy to POD-GP, while reducing computation time by two to three orders of magnitude. Wang *et al.* [146] extended the work by Hesthaven & Ubbiali [143] to time-dependent PDEs and applied it to a quasi-1D PDE problem. In this case, the time coordinate is included as an additional input to the neural network, allowing evaluation of the ROM at different timesteps. For the simple test problem, the authors found ROM accuracy of ∼99% and an acceleration factor of order 10^7^ relative to the FOM. San *et al.* [144] applied the POD-NN approach to the viscous Burgers equation to model time-dependent nonlinear wave propagation. San *et al.* [144] used a different network design from Hesthaven & Ubbiali [143] and Wang *et al.* [146], with San *et al.* [144] building a network that maps from the ROM coefficients at time *t*_*n*_ and any controllable input parameters (e.g. Reynolds number) to an output that characterises the ROM coefficients at time *t*_*n*+1_. Within this framework, they present two variations: (i) a sequential network, where the outputs are the ROM coefficients, and (ii) a residual network, where the outputs are the residual between the ROM coefficients of *t*_*n*+1_ and *t*_*n*_. Of these two approaches, the residual network is found to be superior and both approaches outperform POD-GP for the Burgers equation application. Balzotti *et al.* [147] applied the POD-NN approach to optimal control of steady-state flow in a patient-specific coronary artery bypass graft. The Reynolds number parameterised the inflow and was the single input parameter for which the ROM was constructed. The objective of the optimal control algorithm was to identify the normal stress that has to be imposed at the outlet to ensure a satisfactory agreement between the computed and clinically measured velocity fields. Online evaluation of the ROM took approximately 10^−4^ s, which is a speed-up of order 10^6^ compared to the FOM. The POD-NN model was comparably accurate to a POD-GP model applied to the same problem, but the POD-NN ROM was four orders of magnitude faster [103].

It is also possible to augment POD-GP ROMs with machine learning. Two challenges in POD-GP ROMs are: (i) the potential lack of long-term stability and accuracy and (ii) the lack of complete decoupling for nonlinear governing equation projection onto the reduced basis and the subsequent high cost of evaluating these nonlinear reduced operators. To address the first challenge, Wang *et al.* [148] used a long short-term memory (LSTM) network, a type of recurrent neural network designed to operate on sequential data. The POD coefficients are fed into the LSTM units and the physical/geometric parameters are fed into the initial hidden state of the LSTM. When applied to various problems (3D Stokes flow, 1D Kuramoto–Sivashinsky equation and 2D Rayleigh–Bernard convection), the LSTM-POD-GP ROM is found to improve stability and accuracy compared to POD-GP for nonlinear problems. Furthermore, the LSTM ROM facilitates accurate predictions beyond the time interval of the training data. To address the second challenge, Gao *et al.* [149] proposed a non-intrusive approach to hyper-reduction that approximates the ROM velocity function using a FCNN. The FCNN-enhanced POD-GP ROM was applied to two nonlinear PDEs (1D viscous Burgers equation and 2D flame model) and found to be accurate to approximately 95%. The ROM was also shown to be more stable and accurate for the test problems than POD-GP with alternative hyper-reduction methods (DEIM), in the limit of a small basis. Another approach to improve accuracy is to use machine learning to adapt the ROM to a given input. Daniel *et al.* [150] used a deep classification network to recommend a suitable local POD-GP ROM from a dictionary of possible ROMs. This approach could be used in conjunction with small local ROMs, which have been shown to outperform a single global ROM in terms of accuracy and acceleration [98,151].

#### 3.1.2. Machine learning-based reduced order models

Dimensionality reduction is a crucial step in ROM construction and is commonly performed using techniques such as POD or DMD. Autoencoders (figure 3) are neural networks used to compress and decompress high-dimensional data and are thus being increasingly used in the dimensionality reduction step in reduced models. Autoencoders can provide nonlinear data embedding, whereas POD and DMD offer only a linear reduced basis [34,35]. This could allow autoencoders to compress complex nonlinear data more accurately than POD or DMD. Another approach that can offer nonlinear dimensionality reduction is manifold learning. Csala *et al.* [152] compared four manifold learning (locally linear embedding, kernel principal component analysis (PCA), Laplacian eigenmaps, isometric mapping) and two ML-based (autoencoder, mode decomposing autoencoder) nonlinear dimensionality reduction methods to PCA. They found that all six of the nonlinear dimensionality reduction methods achieved lower reconstruction errors than PCA for spatial reduction, but that only the autoencoder-based reduction was definitively superior for temporal reduction. Maulik *et al.* [34] used a ROM based on a convolutional autoencoder (CAE) and an LSTM to model the viscous Burgers equation and the inviscid shallow-water equations. In these advection-dominated systems, the deep learning (DL)-based ROM outperforms the POD-GP method. The CAE-LSTM approach is 14 times faster than the POD-GP method, producing errors of the same magnitude. Pant *et al.* [35] used a 3D CAE to compress simulation data and advance the solution in time without solving the Navier–Stokes equations in an iterative fashion. Using a 3D CAE allows for features to be extracted in both spatial and temporal axes, which mitigates the need for an additional network (e.g. an LSTM) for time propagation. Using this approach, the authors reduce computational run times by two orders of magnitude compared to traditional CFD solvers.

Fresca *et al.* [153] constructed a POD-DL-ROM that uses POD to reduce the dimensionality of the training data, improve training efficiency and reduce complexity. Compared to previous work by the same authors, enhancing with POD reduces the DL-ROM training time from 15 h to 24 min. The DL-ROM itself uses CAEs and feedforward neural networks trained on the POD-reduced solution vectors. Fresca & Manzoni [145] used the same approach for a series of additional applications including an unsteady advection–diffusion–reaction system, a coupled PDE–ODE Monodomain/Aliev-Panfilov system, a nonlinear elastodynamics problem and the unsteady Navier–Stokes equations. For the most pertinent example, the Navier–Stokes problem, the acceleration factor was of the order 10^5^ compared to the FOM while achieving a comparable accuracy to the more expensive non-enhanced DL-ROM. Fresca & Manzoni [154] used the same POD-DL-ROM for flow around a cylinder, FSI between an elastic beam and a laminar flow, and blood flow in a cerebral aneurysm. High levels of accuracy are qualitatively displayed for each application. Acceleration factors for all applications are of the order of 10^5^. Essentially, the approach of Fresca *et al.* [145,153,154] reduces the size of the data passed through the network and the amount of training parameters required, thus improving the efficiency of training and testing while preserving the precision of the DL-ROM without POD enhancement.

#### 3.1.3. Conclusion

Machine learning (ML) has a lot to offer the ROM field, as demonstrated by the various studies in table 5 that used ML and ROMs in conjunction. ML can be used to provide closure in projection-based ROMs, improve interpolation in POD-Interpolation ROMs, improve long-time ROM predictions, or offer alternative dimensionality reduction algorithms that are essential in almost all ROMs. ML-ROMs are able to address the weaknesses that hinder various reduced order methods, such as poor performance for nonlinear problems, lack of stability or lack of generality. As a result, ML-ROMs will typically be suitable for a wider array of vascular flow problems than the traditional ROM techniques from which they are derived. Balzotti *et al.* [147] demonstrated the superior acceleration capacity of a POD-NN ROM compared to a POD-GP ROM for a vascular flow problem due to the POD-NN approach being better suited for the nonlinear nature of the problem. Similarly, Csala *et al.* [152] demonstrated the superior spatial reduction capability of nonlinear ML-based dimensionality reduction techniques when applied to aneurysm blood flow, which suggests that more accurate models may be possible using ML-based reduction techniques. Fresca & Manzoni [154] conversely used traditional dimensionality reduction techniques (POD) in conjunction with an ML-based ROM and achieved high levels of accuracy and acceleration for aneurysm blood flow. While not for vasclar flow applications, Wang *et al.* [148] and Gao *et al.* [149] augmented POD-GP ROMs with ML and achieved improved stability and accuracy. These findings demonstrate that ML-ROMs are a compelling option for vascular flow problems. In particular, ML-ROMs can offer methods suitable for vascular flow problems that are nonlinear, geometrically complex, multi-physics and multi-scale in time.

**Table 5.** Machine learning ROM studies for various applications.

reference	method	application	comments on accuracy and/or acceleration
*ML-augmented ROMs*			
Hesthaven & Ubbiali [143]	POD-NN	parameterised steady-state PDEs (Poisson equation, LDC)	POD-NN achieves similar accuracy to POD-GP while reducing CPU time by 2–3 orders of magnitude
Wang *et al.* [146]	POD-NN	parameterised unsteady PDE (quasi-1D CVRC flow)	accuracy of ∼99% and acceleration factor of 10^7^
San *et al.* [144]	POD-NN (SN and RN)	viscous Burgers equation (time-dependent nonlinear wave propagation)	POD-NN approach outperforms POD-GP in interpolation and extrapolation and is 10^2^ times faster
Balzotti *et al.* [147]	POD-NN	steady-state flow in a coronary artery bypass graft	POD-NN achieves similar accuracy to POD-GP and speed-up of 10^6^ and 10^4^ relative to FOM and POD-GP, respectively
Wang *et al.* [148]	LSTM-enhanced POD-GP	3D Stokes flow, 1D Kuramoto–Sivashinsky equation, 2D Rayleigh–Bernard convection	ROM improves stability and accuracy of POD-GP for nonlinear problems and allows time predictions beyond training data
Gao *et al.* [149]	FCNN-enhanced POD-GP	nonlinear PDEs (1D viscous Burgers equation and 2D flame model)	ROM accuracy is ∼95%. ROM is more stable and accurate than POD-GP with DEIM (in the small basis limit)
*ML-based ROMs*			
Maulik *et al.* [34]	CAE-LSTM	viscous Burgers equation and shallow water equations	CAE-LSTM has similar accuracy to POD-GP and is ∼14 times faster
Pant *et al.* [35]	3D CAE	2D flow (past a circular/square cylinder, over a plate, in a channel) and SST data	reconstruction accuracy is good and model can predict future timesteps accurately. Acceleration factor of 10^2^
Fresca *et al.* [153]	POD-enhanced CAE NN	left ventricular cardiac electrophysiology	POD enhancement reduces training time from 15 h to 24 min
Fresca & Manzoni [154]	POD-enhanced CAE NN	flow around cylinder, FSI of beam and laminar flow, cerebral aneurysm flow	high levels of accuracy are displayed and acceleration factors are of order 10^5^ for all applications
Fresca & Manzoni [145]	POD-enhanced CAE NN	flow past a cylinder	POD-enhanced ROM has similar accuracy to non-enhanced DL-ROM. Acceleration factor is 10^5^

CAE, convolutional autoencoder; CPU, central processing unit; CVRC, continuously variable resonance combustor; DEIM, discrete empirical interpolation method; DL, deep learning; FCNN, fully connected NN; FOM, full-order model; GP, Galerkin projection; LDC, lid-driven cavity; LSTM, long short-term memory; ML, machine learning; NN, neural network; PDE, partial differential equation; POD, proper orthogonal decomposition; RN, residual network; ROM, reduced order model; SN, sequential network; SST, sea surface temperature.

### 3.2. Physics-informed machine learning simulation

Machine learning can be used to construct fast surrogate models for vascular flow problems that directly predict haemodynamic quantities of interest, as in work by Itu *et al.* [37], Rutkowski *et al.* [155] and Liang *et al.* [156] (discussed further in §3.3.1). A criticism of this approach is that the models do not guarantee the underlying physics in the problem will be respected. This can be somewhat resolved by incorporating known physics into the learning procedure [157]. The most widely used techniques to achieve this are physics-informed neural networks (PINNs), which can combine data acquired from simulations or experiments with knowledge of the underlying governing equations and boundary conditions [36,158]. In contrast to most machine learning simulation techniques, PINNs can be used in the absence of data. PINNs without training data may be less accurate than with data, but data-free PINNs offer a direct alternative to standard numerical techniques [159]. While PINNs were initially developed for solution and discovery of PDEs in forward and inverse scenarios, the development of data-free and parametric PINNs has since seen them applied to simulation acceleration. PINNs have been demonstrated to vastly reduce simulation times, particularly in the context of parametric design optimisation problems, hence our focus on this technique in this review [160,161].

A typical PINN is shown in figure 3. The PINN consists of a network with simulation parameters (e.g. space/time coordinates) as input and solution fields (e.g. velocity/pressure) as output. Fully connected neural networks are typically used for PINNs, but various other approaches have demonstrated superior results for certain applications [162]. For the chosen architecture, automatic differentiation is typically used to differentiate network outputs with respect to its inputs, thus acquiring derivatives such as *u*_*x*_, *p*_*x*_, *u*_*t*_, etc. which can be combined to formulate governing equation residuals. For the incompressible Newtonian Navier–Stokes equations, the residual of the *x*-momentum equation will take the form

3.1 $$F_{1}=u_{t}+uu_{x}+vu_{y}+wu_{z}+p_{x}-\frac{1}{\text{Re}}(u_{xx}+u_{yy}+u_{zz}),$$

where ***u*** = (*u*, *v*, *w*) is velocity, *p* is pressure and Re is the Reynolds number. Reduced Navier–Stokes equations (e.g. equation (2.3) for 1D blood flow) can also be used as residuals [163]. The residuals are included in the loss function for the network, which encourages the network to learn mappings that minimise the residuals and therefore satisfy the underlying governing equations. It is possible to enforce additional loss constraints that penalise the network for non-satisfaction of boundary conditions, such as the no-slip condition that is often applied on blood vessel walls. Alternatively, boundary conditions can be imposed as hard constraints through the network architecture [164]. Once trained, the PINN is able to infer solution fields that satisfy data, governing equations and boundary conditions.

PINNs are designed to improve the efficiency of non-informed networks through reducing the amount of data required and helping the network train efficiently by discarding non-physical mappings. A further benefit of PINNs is their potential to be used as an alternative to traditional numerical solvers. If data are unavailable, PINNs can be trained on PDE residual points and boundary conditions alone, mirroring traditional numerical techniques’ procedure. However, the input coordinates need only be a point cloud rather than the volumetric mesh required for typical numerical solvers. Furthermore, unlike traditional numerical solvers, when a problem is ill-posed with incomplete or noisy boundary conditions, PINNs are still a viable option [165]. A final benefit of PINNs is that they are well suited to solving inverse problems as well as forward problems, whereas traditional numerical techniques are usually only suitable for forward problems.

Once trained, a PINN can quickly infer physics-respecting solution fields given spatio-temporal inputs, making them a promising acceleration technique. However, generalising a PINN for additional input parameters can decrease accuracy and increase training time, so the fast inference speeds must be balanced against training cost and accuracy. Despite their promise, PINNs are a relatively new technique for simulation and the application of PINNs towards acceleration and vascular flow is in its infancy. We aim to address three questions in order to determine the usefulness of PINNs for vascular flow acceleration: (i) How suitable are PINNs for simulation acceleration? (ii) How fast are PINNs relative to traditional numerical techniques? (iii) Are PINNs suited to the complexities of vascular flow acceleration?

#### 3.2.1. How suitable are physics-informed neural networks for acceleration?

Developing and using a PINN model often consists of three stages: (i) generating or acquiring data from simulations or experiments, (ii) training the network while incorporating known physics and boundary conditions and (iii) using the model to infer solutions for new inputs. In inference mode, PINNs are usually faster than a traditional numerical model applied to the same problem. However, if the PINN relies on data generated by the numerical model and requires a potentially expensive training procedure prior to use, then the question of how to use PINNs for acceleration remains. In order to prove a useful and powerful tool for simulation acceleration, PINNs will either need to be able to generalise to unseen problems in a similar fashion to how parametric ROMs operate, or they will need to have a sufficiently small training time such that training a new PINN model is more efficient than solving a traditional numerical model.

Generalising a PINN model can require adding additional parameters into the training procedure. These parameters could describe geometry, boundary conditions, or material properties and there are various ways to incorporate this information into the PINN. The most straightforward approach is to include additional network input parameters. Arthurs & King [160] introduced two input parameters describing the peak inflow rate and diameter in a pipe flow problem. Sun *et al.* [159] similarly included parameters that describe geometry and viscosity as input to their PINN. When parameterising the network in this manner, an active learning strategy can reduce the cost of up-front data generation. This consists of refining the training data with additional finite-element model (FEM) samples in regions of the parameter space where the PINN prediction is poor. Costabal *et al.* [167] used a positional encoding mechanism for PINNs that creates an input space for the network representing the geometry of a given object, improving PINN performance in complex geometries. However, for a Poisson forward problem in a simple domain, the positional encoding method was not observed to outperform traditional PINNs. De Avila Belbute-Peres *et al.* [168] developed a hyper-PINN approach, where an additional network is trained on sets of model input parameters (e.g. geometric parameters, boundary conditions, material properties) and network weights from previously trained PINN models for each simulation configuration. This precursor network learns how to map from the input parameter space to the weights needed for the PINN model for that particular parameter configuration. For a new parameter set, the precursor produces the weights needed to directly use the PINN in inference mode, thus bypassing the need to train a new PINN model entirely.

Alternatively to generalizing PINNs, reducing training time sufficiently can mean that training a new PINN for each problem is still a tractable approach. Kissas *et al.* [163] suggested transfer learning to solve this problem. Transfer learning consists of initialising new PINN models with the parameters from a model previously trained on a similar problem, which can drastically reduce training time. This is similar to providing an accurate initial guess in iterative numerical methods. A transfer learning approach could allow for a new PINN to be trained for each new simulation configuration (new geometry, boundary conditions, etc.) while still providing an acceleration relative to solving the problem with traditional numerical techniques. For this approach to make sense, the new PINN must be trained without the use of training data from solving the numerical model. To this end, Desai *et al.* [168] proposed a one-shot transfer learning approach for PINNs, which consists of training for a selection of PDEs and then reusing some of the trained layers for an unseen PDE, thereby reducing training time. Another approach to accelerate training is to incorporate a hyper-parameter into the activation functions in the PINN [169]. The hyper-parameter dynamically changes the loss function topology throughout training and is shown to accelerate PINN convergence and increase accuracy. Residual-based adaptive refinement can also accelerate training [170,171]. This approach aims to increase the number of network training points in regions where the PDE residual is inaccurate throughout training, thus accelerating convergence.

#### 3.2.2. How fast are physics-informed neural networks?

Once the PINN training time is sufficiently reduced, or the network is generalized appropriately, the question of how fast PINNs are relative to traditional numerical techniques remains. Table 6 collates the literature on PINNs where the authors commented on the acceleration offered by their approach.

**Table 6.** Various PINN papers that mention the acceleration capability of their method.

reference	method	application	comments on accuracy and/or acceleration
*general applications*			
Hennigh *et al.* [161]	PINN	heat sink design optimisation problem	total compute time is reduced by approximately 45 000 times and approximately 150 000 times compared to commercial and OpenFOAM solvers, respectively
Arthurs & King [160]	PINNs with active training	parametric Navier–Stokes (two parameters)	PINN parameter sweep takes 7.6 s compared to 54 min for FEM. 400 times faster
*cardiovascular applications*			
Gao *et al.* [149]	PI-CNN	SR of parameterised flow fields for idealised vascular problems	model accurately refines spatial resolution by 400 times and provides speed-up of 3364 times relative to CFD model
Buoso *et al.* [172]	PINNs with RBF reduction	left-ventricular biophysical modelling	30 times faster than FEM including training (for evaluating only one condition). Accuracy for ejection fraction 97%, peak SBP 93%, stroke work 96%, myocardial strains 86%
Sun *et al.* [159]	PINNs	parametric flow in 2D idealised stenotic and aneurysmal vessels	PINN evaluation is 2000 times faster than CFD model, but training takes hundreds of times longer than individual CFD simulations

CFD, computational fluid dynamics; FEM, finite-element model; PI-CNN, physics-informed convolutional neural network; PINN, physics-informed neural network; RBF, radial basis functions; SBP, systolic blood pressure; SR, super-resolution.

Arthurs & King [160] and Hennigh *et al.* [161] conducted design optimisation studies using PINNs. Arthurs & King [160] developed a parametric PINN model for Navier–Stokes applications and ran a parameter sweep experiment to identify the value of the geometric input parameter that would lead to a target pressure drop. This is a typical many-query problem, where repeated model evaluations are required to identify some kind of threshold in the output variable. The trained PINN required only 7.6 s to perform the sweep over 81 parameter points, whereas the same sweep using FEM would have taken 400 times longer. Scaling up the number of parameter queries to 1 million only increases the run time to 11.1 s, highlighting the scalability of the PINN due to its fast inference speed. However, it should be noted that the PINN evaluation was only performed at two spatial points, as this is all that is required to calculate the pressure drop. This demonstrates a benefit of PINNs, in that they can be used to query specific regions of interest, but the FEM model inherently evaluates the entire spatial field, so directly comparing model efficiency is not fair in this case. Hennigh *et al.* [161] presented NVIDIA SimNet, an AI-accelerated multi-physics simulation framework based on PINNs. They studied a design optimisation problem where SimNet is able to reduce total compute time by approximately 45 000 times compared to a commercial solver and 150 000 times compared to OpenFOAM. Gao *et al.* [173] trained physics-informed CNNs for super-resolution of low resolution flow field inputs using only knowledge of the conservation laws and boundary conditions. They applied this approach to 2D flow in a vascular domain and parametric super-resolution for internal flow with a parameterised inlet velocity profile. The model accurately refines the spatial resolution by 400 times for the flow fields with any new inlet BCs sampled in the 20-dimensional parameter space. The speed-up time for the trained model compared to the highly resolved CFD model is 3364 times. Sun *et al.* [159] used data-free parametric PINNs for flow in 2D idealised stenotic and aneurysmal vessels. They achieved accurate results in all test problems with mean test errors of order 10^−4^–10^−8^ depending upon the problem and variable of interest. The authors noted that in the data-free PINN regime, implementing boundary and initial conditions with hard constraints improved performance when compared with the more widely used soft constraints. The trained PINN can be evaluated in 0.02 s, whereas the CFD model takes 40 s, yielding a speed-up of 2000 times. However, training the PINN took hundreds of times longer than an individual CFD simulation. The PINN will therefore only reduce total computational cost in scenarios where a large number of model evaluations are required, such as uncertainty quantification or design optimisation. Sun *et al.* [159] suggested that the speed-up offered by their approach will be increasingly advantageous when more complex applications are considered.

#### 3.2.3. Physics-informed neural networks for vascular flow acceleration

PINNs are inherently suited to nonlinear problems due to the nonlinear function approximating capacity of the network. In fact, the earliest applications of PINNs include nonlinear PDEs, such as the Navier–Stokes and Schrödinger equations [36]. Since then, PINNs have been successfully applied to various cardiovascular fluid dynamics problems, all of which are governed by the nonlinear Navier–Stokes equations [162,163,174–178].

Individual complex geometries are relatively straightforward to handle with PINNs. Instead of the usual volumetric mesh required for traditional numerical techniques, PINNs require only spatio-temporal coordinates as input and do not require connectivity between these points. Volumetric meshes may still be required in order to generate simulation data to train the PINN, but if the PINN is used to generalise across geometries, then users can forego the time-consuming meshing step for some of the geometries [159]. Raissi *et al.* [179] used PINNs to infer flow fields from concentration fields in an image-derived 3D aneurysm model and Sun *et al.* [159] applied PINNs with hard boundary condition enforcement to model flow in idealised stenosis and aneurysm models. This highlights two geometrically relevant applications of PINNs.

PINNs can also tackle multi-physics problems. Figure 3 shows a single-physics PINN, but additional physics can be added by using a second network that maps from the same inputs as the first network (space and time) to different outputs (e.g. displacements and stresses for solid mechanics). It is therefore possible to calculate all the required derivatives in order to impose the governing equations and boundary conditions from each aspect of the multi-physics problem. This approach has been applied to an inverse Navier–Stokes and Cahn–Hilliard blood flow-thrombosis problem [177], multi-phase heat transfer [180] and FSI [181].

Basic PINNs are not commonly applied to extrapolating the associated PDE in time. Kim *et al.* [182] proposed a dynamic pulling method (DPM) to overcome this issue. DPM manipulates the PINN’s gradients to ensure the PDE’s residual loss term continuously decreases during training. This is shown to improve extrapolation in time for various test problems. Basic PINNs are also not well suited to problems spanning very large spatial regions. This issue with large spatial and temporal domains is that the domain can become arbitrarily large, leading to prohibitive training times. The primary approach to tackling these problems is incorporating domain decomposition into the PINN framework. Decomposing the large spatio-temporal domain into smaller sub-domains allows for sub-PINNs to be trained in each sub-domain. This improves training efficiency as well as reducing error propagation, allowing for domain-specific hyper-parameter tuning, increasing representation capacity and facilitating paralellisation [183].

Conservative PINNs (cPINNs), extended PINNs (XPINNs) and parallel-in-time PINNS (PPINNs) are three possible domain decomposition approaches that can tailor PINNS for multi-scale problems. cPINNs enforce conservation properties at spatial sub-domain boundaries using flux continuity and solution averaging across the interfaces [183]. XPINN is an extension to cPINN that applies to any type of PDE, not only conservation laws, and allows for decompositions in time and space [184]. Shukla *et al.* [185] compared cPINN and XPINN for a series of forward problems and found that for space decomposition, cPINNs are more efficient in terms of communication cost but that XPINNs are more flexible as they can handle time decomposition, a wider array of PDEs and arbitrarily shaped sub-domains. PPINNs are an extension to PINNs that mitigate the issue of long-time integration through time-domain decomposition and using a coarse-grained solver for long-time supervision [186]. The coarse-grain solver provides initial conditions for the PPINN in each time sub-domain. The coarse-grain solver needs be fast enough to solve the long-time PDE with some degree of accuracy cheaply, hence reduced-order or simplified models are viable options. Meng *et al.* [186] stated that the PPINN method could be extended to spatial domain decomposition, with a coarse-grained solver used to estimate the global solution and then a series of PINNs applied in parallel to spatial sub-domains, thus increasing training efficiency relative to applying one PINN for the entire domain.

#### 3.2.4. Conclusion

PINNs offer a mixture of numerical mechanistic models and data-driven phenomenological models. Training a PINN model can be expensive compared to running a high-fidelity numerical model, so they are most useful for acceleration when a once-trained PINN can be used for numerous parameter or geometry instances. Various methods have been studied to parameterise PINNs [159,160,166,167]. An alternative approach is to use PINNs in conjunction with transfer learning techniques to quickly retrain the model for a new system instance [168]. Employing techniques such as these can make PINNs a viable option for accelerating vascular flow simulations, particularly as PINNs (and extensions thereof) are well suited to handling nonlinear, geometrically complex, multi-physics and multi-scale modelling problems.

### 3.3. Other techniques

Given the relatively recent application of machine learning to simulation and the continued growth of the machine learning field, there are numerous other machine learning methods that have been or can potentially be applied to vascular flow acceleration. Reviewing them all in detail is beyond the scope of this study, and in most instances, there is insufficient relevant literature to do so, but we will briefly discuss several of these approaches and highlight how they may prove useful in the future for our target application.

#### 3.3.1. Physics-agnostic machine learning simulation

An alternative to augmenting/constructing ROMs using machine learning or attempting to encode physics into machine learning is to build a machine learning model that directly predicts the haemodynamic quantities of interest from inputs such as images or geometries [37,155]. Some of these approaches are collated in table 7. One of the earliest examples of this is by Itu *et al.* [37], who used a machine learning model to predict FFR given parameterised coronary artery anatomy as input. The model consists of a FCNN with inputs corresponding to features of the coronary anatomy and FFR as the solitary output. Using this approach, the authors achieved an accuracy of 83.2% in correctly diagnosing positive ischaemia and reduced model run time by a factor >80.

**Table 7.** Various machine learning simulation papers applied to vascular flow problems that mention the acceleration capability of their method.

reference	method	application	comments on accuracy and/or acceleration
*general applications*			
Cai *et al.* [187]	DeepONet	steady-state electroconvection	accuracy >99%. Acceleration factor approximately 10^3^
Mao *et al.* [188]	DeepONet	coupled flow and finite-rate chemistry	MSE is approximately 10^−5^. Acceleration factor approximately 10^5^
*cardiovascular applications*			
Itu *et al.* [37]	FCNN	FFR prediction from coronary artery anatomy	83.2% diagnostic accuracy for ischaemia. Acceleration factor >80
Liang *et al.* [156]	AE and FCNN	steady-state haemodynamics prediction in thoracic aorta	velocity accuracy, 98.0%. Pressure accuracy, 98.6%. Acceleration factor approximately 900
Morales *et al.* [189]	FCNN	ECAP prediction from LAA geometry	mean accuracy, 95.3%. Acceleration factor 144^a^
	FCNN with PCA		mean accuracy, 94.8%. Acceleration factor 7200^a^
Ferdian *et al.* [190]	residual CNN	super-resolution of aortic 4D flow MRI	flow rate prediction accuracy >95%. Prediction time 40–90 s
Gharleghi *et al.* [191]	U-Net-style CNN	transient WSS prediction in left main bifurcation of coronary arteries	accuracy >95%. Prediction time of 0.2 and 0.001 s with CPU and GPU, respectively^c^
Li *et al.* [38]	Point-Net	haemodynamics prediction before and after coronary artery bypass surgery	prediction accuracy ∼90%. Acceleration factor 600
Li *et al.* [39]	Point-Net	haemodynamics prediction before and after aneurysm treatment by FDS	prediction accuracy >87%. Acceleration factor 1800
Yin *et al.* [192]	DeepONet	predicting damage progression and *P*–*V* curves in aortic dissection	*P*–*V* accuracy $>95{\%}^{b}$. Prediction time is <1 s, FOM simulation time is ∼12 hours using 20 processors

^a^Ten-fold cross-validation used with 300 geometries. One round of cross-validation on 30 geometries took 30 s or 25 min for each model. This is used to calculate evaluation time for one geometry and compared to reported 2 h CFD simulation time to calculate acceleration factors.

^b^*P*–*V* accuracy taken for test cases with damage included, from table 3 of [192].

^c^Network requires steady-state CFD result as input, which takes <2 min to calculate. With this included, acceleration factor is approximately 90.

AE, autoencoder; CFD, computational fluid dynamics; CPU, central processing unit; DeepONet, deep operator network; ECAP, endothelial cell activation potential; FCNN, fully connected NN; FDS, flow-diverting stent; FOM, full-order model; FFR, fractional flow reserve; GPU, graphics processing unit; LAA, left atrial appendage; MRI, magnetic resonance images; MSE, mean-squared error; NN, neural network; PCA, principal component analysis; *P*–*V*, pressure–volume.

Liang *et al.* [156] trained a DNN to predict steady-state pressure and velocity fields in the thoracic aorta using 729 aorta geometries generated from a statistical shape model and CFD data generated for each geometry [193]. The DNN consisted of autoencoders to encode the aorta shapes and the fields of interest and another network to map between the encoded shapes and fields. The trained network predicted velocity and pressure fields with mean errors of 2.0% and 1.4%, respectively. DNN evaluation time is approximately one second, whereas each CFD simulation took approximately 15 min, giving a speed-up of approximately 900 times. Liang *et al.* [194] applied this network structure to identifying the geometry corresponding to a particular pressure field, thus demonstrating an application of this method to inverse modelling. Morales *et al.* [189] applied two FCNNs, one with prior dimensionality reduction and one without, to predict endothelial cell activation potential (ECAP) from left atrial appendage (LAA) geometry. Their models were trained on 210 LAA geometries using CFD data. With and without dimensionality reduction, the average error was 5.8% and 4.7%, respectively. The network with dimensionality reduction was approximately 50 times faster than the other network when performing cross-validation. Gharleghi *et al.* [191] used a machine learning surrogate to replace a transient CFD solver in order to calculate WSS in the left main bifurcation of the coronary artery. The network requires the steady-state CFD solution for a given case as an input, but can then predict the transient WSS to an accuracy of >95% within 0.2 s using a CPU and 0.001 s using a GPU. Rutkowski *et al.* [155] trained a CNN to map from 4D flow phase-contrast magnetic resonance images to highly resolved flow fields using CFD data as labels. The focus of this work was fast and accurate flow field generation directly from images, foregoing the need for time-consuming and expensive simulation set-up and execution. The network successfully de-noised flow images, improved velocity field accuracy and enhanced near-wall flow measurements. Ferdian *et al.* [190] similarly developed a residual network that was applied to super-resolution of 4D flow magnetic resonance images of aortic blood flow. Their approach was able to predict flow rates in a real patient to greater than 95% accuracy within 40–90 s depending on the image size.

Various physics-agnostic machine learning simulation methods have been able to accurately and efficiently predict flow fields and flow-derived quantities in vascular flow applications. Provided that a FOM can be constructed and that sufficient data can subsequently be generated, the breadth of vascular flow problems that could be accelerated by these surrogate models is large. However, the vast amount of data required to generate accurate results could constrain these approaches, particularly in vascular flow applications where geometric data are typically derived from medical images that can be expensive to acquire and difficult to process. This is highlighted by Liang *et al.* [156], Morales *et al.* [189] and Gharleghi *et al.* [191] relying upon data augmentation strategies to extend their cohorts of real patients into larger cohorts of mostly synthetic patients. While this is necessary to create sufficiently large datasets, there is a risk that the augmentation may produce unrealistic results, as demonstrated by Morales *et al.* [189] discarding 30% of their initial training samples due to unrealistic flow features. It is possible that data augmentation approaches from the wider machine learning field, such as variational autoencoders or generative adversarial networks, could provide techniques to generate highly realistic synthetic datasets [195–197]. Another issue with physics-agnostic machine learning simulation methods is that the up-front cost of running CFD simulations in large cohorts to generate training data and the subsequent cost of training the complex network can lead to large overall costs. Despite these challenges, machine learning surrogate models are able to make predictions in previously unseen geometries due to being trained over an extensive array of different geometries. This is a crucial challenge in many vascular flow modelling problems that most acceleration techniques do not address with such generality.

#### 3.3.2. Point network simulation

Typical convolutional deep learning architectures require regular input data, such as images. Point-Net was developed to allow the direct use of irregular point cloud data with techniques typically applied to regular input data [198]. A benefit of using a Point-Net architecture is its ability to generalise well to new input point clouds. This means generalising to unseen geometries for vascular flow applications, which can lead to large savings in simulation times. Point-Net-based models have been applied to cardiovascular flow problems. Li *et al.* [38] used a Point-Net-based model to predict steady-state haemodynamics before and after coronary artery bypass surgery. Their approach yielded a prediction accuracy for velocity and pressure fields of around 90%. The time to evaluate the deep learning model was 600 times less than for the CFD model (1 s versus 10 min), although 40 h of training time was required prior to using the former. The same authors also applied their Point-Net-based model to predict steady-state aneurysm haemodynamics before and after treatment with a porous-medium flow-diverting stent model [39]. A similar prediction accuracy was found (>87%) and the calculation time was reduced by a factor of 1800. Kashefi & Mukerji [199] developed a physics-informed Point-Net (PIPN) and evaluated it for steady-state incompressible flow problems. The acceleration factor is approximately 35 for trained PIPN evaluation compared to the standard numerical solver. Compared to PINNs, the accuracy of PIPNs is similar when trained to the same convergence criterion, but the computational cost of PINNs is 18 times greater. This factor is increased when exploiting the inherent generalisation of PIPN to model new geometries, as in this scenario, the PINN will often need to be re-trained. PIPN is a recent technique that has not yet been applied to vascular flow.

#### 3.3.3. Operator networks

The function approximation capacity of neural networks is well known, but it is also possible for neural networks to approximate operators that map between function spaces [200]. The first and most general operator network is the deep operator network (DeepONet) [40]. DeepONet consists of a branch network, which encodes the input function space, and a trunk network, which encodes the domain of the output functions. The input to the branch network are function values at fixed sensors and the input to the trunk network are spatio-temporal coordinates at which to evaluate the operator. The output of the trunk network is a set of basis functions, and the output of the branch network is the basis coefficients [41]. Combining the basis coefficients and functions using the dot product gives the operator network output. Following training, the DeepONet approximates the underlying solution operator for the input function and coordinate spaces. Other operator learning methods include the Graph Kernel Network and Fourier Neural Operator [201,202]. Physics-informed extensions to operator networks that can reduce the required training data have also been studied [41,203].

Operator learning approaches have been applied to various linear and nonlinear problems involving explicit and implicit operators [40]. Cai *et al.* [187] used DeepONets for electroconvection, which is a multi-physics problem involving coupled flow, electric and concentration fields. They noted that training the DeepONets takes approximately 2 h, but the evaluation time once trained is less than 1 s, representing a speed-up of approximately 1000 times when compared with the NekTar solver used to generate training data. Mao *et al.* [188] used DeepONet for a hypersonic flow problem involving a coupling between flow and finite-rate chemistry. They found that the trained network was five orders of magnitude faster than the CFD solver used to generate the data. Furthermore, Cai *et al.* [187] and Mao *et al.* [188] combined multiple DeepONets to build a DeepM&MNet, which is specifically designed to handle multi-scale and multi-physics modelling. DeepONets have also been used as a surrogate for expensive microscopic models, thus accelerating the coupling between micro- and macro-scale models [204]. Recent work has also investigated using physics-informed DeepONets for long-time integration of parametric partial differential equations [205]. Applications of operator learning to vascular flow problems are limited, but two examples are by Yin *et al.* [192] and Arzani *et al.* [206]. Yin *et al.* [192] applied DeepONets to simulation of aortic dissection, a complex fluid–structure interaction problem. The DeepONet was able to make predictions in less than 1 s, whereas the FEM used to produce training data took approximately 12 h to run using 20 processors. Arzani *et al.* [206] applied an operator learning surrogate model to 2D cardiovascular flow applications, but the focus of this work was on the interpretability and generalisation rather than acceleration.

Compared to function-based learning strategies, a benefit of operator learning is that they demonstrate small generalisation errors [40]. Furthermore, DeepONets have been shown to overcome the curse of dimensionality, in that they do not require exponentially more training data to improve the approximation accuracy [207]. These techniques can potentially address many of the inherent complexities of vascular flow, particularly the multi-physics and multi-scale nature of the problem, but they have not yet seen widespread adoption.

## 4. Discussion and outlook

### 4.1. Summary

This review presents simulation acceleration methods based on ROM and machine learning for the target application of vascular flow. The review focuses on five complexities that are common in vascular flow problems, but which are also found across a multitude of other domains; namely: (i) nonlinearity, (ii) geometric complexity, (iii) multi-physics, (iv) multi-scale in time and (v) multi-scale in space. Each complexity presents unique challenges for vascular flow simulations and their acceleration. The ROM methods discussed in this review are spatial dimension reduction (SDR), POD and dynamic mode decomposition (DMD) ROMs, as well as brief overviews of reduced basis (RB) methods and proper generalised decomposition (PGD). The machine learning approaches reviewed are machine learning-augmented ROMs, machine learning-based ROMs, physics-informed neural networks (PINNs), physics-agnostic networks, Point-Nets and operator networks. We found that all acceleration methods are well suited to some of the complexities of vascular flow and limited for others, as highlighted in table 8.

**Table 8.** Reduced order modelling and machine learning acceleration methods and their suitability for modelling various vascular flow complexities. RB, PGD and Point-Net simulation acceleration approaches were briefly reviewed in this paper but not in sufficient detail to include in this table.

method	nonlinearity	geometric complexity	multi-physics	multi-scale (time)	multi-scale (space)
*ROMs*					
SDR	✓	✗	∼	✗	✓
POD-P	∼	✓	✓	✗	✗^a^
POD-I	✓	✓	✓	✗	✗^a^
DMD	✓	✓	∼	✓	✗^a^
*machine learning-augmented ROMs*					
POD-I-NN^b^	✓	✓	✓	∼	✗^a^
POD-P-NN^b^	✓	✓	✓	∼	✗^a^
*machine learning methods*					
physics-agnostic	✓	✓^c^	✓	✓^d^	✓^d^
PINN	✓	✓	✓	✓^e^	✓^e^
DeepONet	✓	✓	✓	✓	✓

Key: ✓, method is suitable; ∼, somewhat suitable; ✗, not suitable.

^a^In isolation the methods are not well suited for spatial multi-scale problems, but they can be coupled to patient-specific SDR models so that boundary conditions are derived from large portions of the vasculature.

^b^Includes various types of NN used in conjunction with the ROM approach, such as FCNNs or RNNs.

^c^Physics-agnostic approaches are not only suitable for complex individual geometries, but are capable of generalising to previously unseen geometries.

^d^While suitable for multi-scale problems in principle, the data-hungry nature of physics-agnostic approaches may lead to prohibitive data requirements for problems spanning large spatial and time scales.

^e^Basic PINNs are not designed for multi-scale problems, but extensions such as cPINNs, XPINNs and PPINNs are.

cPINNs, conservative PINNs; DeepONet, deep operator network; DMD, dynamic mode decomposition; FCNN, fully connected NN; PGD, proper generalised decomposition; POD, proper orthogonal decomposition; POD-I, POD-Interpolation; POD-P, POD-Projection; NN, neural network; PINN, physics-informed NN; PPINNs, parallel-in-time PINNs; RB, reduced basis; RNN, recurrent NN; ROM, reduced order model; SDR, spatial dimension reduction; XPINNs, extended PINNs.

#### 4.1.1. Reduced order modelling

SDR methods are suitable for capturing spatial multi-scale behaviour and some nonlinear and multi-physics effects, but only in simplified geometries where axisymmetry or other assumptions are valid [45]. These methods calculate bulk quantities instead of full spatio-temporal fields and are not designed for temporal multi-scale problems. SDR methods are widely used in various vascular applications, with one of its most common uses in deriving boundary conditions for 3D models [45,63]. Due to their simplistic nature, SDR models can provide large acceleration ranging from two to six orders of magnitude [54].

POD-based ROMs branch into two categories depending upon whether they combine POD with projection or interpolation. POD-Projection and POD-Interpolation ROMs are able to calculate 3D time-varying solution fields in individual complex geometries. POD-Projection has been applied to various vascular flow problems [30,80–82,92,100,101]. Both approaches are suitable for multi-physics problems. For nonlinear problems, the projection applied to the governing equations does not fully de-couple the ROM and the full-order model, limiting the acceleration offered by POD-Projection ROMs. POD-Interpolation does not depend upon the governing equations of the system, so it does not suffer the same limitations for nonlinear applications. However, POD-Interpolation ROMs have been shown to generalise less effectively than their projection-based counterparts [96]. Neither POD-Projection nor POD-Interpolation are well suited to multi-scale modelling in time, with the long-term stability of POD modes not guaranteed. Finally, while neither approach is inherently well suited to spatial multi-scale modelling, coupling the POD-based ROM to an SDR ROM could produce a model that can quickly and accurately provide full spatio-temporal fields in a region of interest while capturing the influence of the systemic vasculature. Due to the non-iterative nature of POD-Interpolation, it can typically provide large accelerations ranging from two to six orders of magnitude, whereas POD-Projection acceleration ranges from one to three orders of magnitude [77,97,99,100].

Similarly to POD-based ROMs, DMD ROMs can provide full spatio-temporal fields in individual geometries and could be coupled to SDR models to capture the influence of large regions of the vasculature. DMD ROMs are less common than POD-based approaches, so application to multi-physics simulation acceleration has not been thoroughly investigated. The main benefit to DMD ROMs is that they are designed to approximate the temporal dynamics of the system, which makes them well suited to the long-time model integration required in temporal multi-scale problems.

Other techniques include RB methods and PGD. RB methods are a similar approach to POD-Projection ROMs and have been successfully applied to various nonlinear, multi-physics, geometrically complex problems [126–128]. RB methods have been applied to vascular flow problems such as flow field calculation in 2D parameterised carotid arteries, inverse modelling in stenosed arteries and flow in femoropopliteal bypass problems [123–125]. The acceleration offered by RB methods ranges from two to three orders of magnitude. PGD sits apart from most ROM methods, as it uses separated representations and successive enrichment *a priori* instead of applying dimensionality reduction to snapshots from the full-order model *a posteriori* in order to construct the reduced basis [129]. PGD has been applied to Navier–Stokes and rheology applications with acceleration ranging from one to two orders of magnitude [131,133]. This approach is well suited for separable problems, whether the separation is in space or time [130,134,135]; however, it has not been applied as widely as other ROM methods and has seen no application to vascular flow simulation acceleration.

#### 4.1.2. Machine learning simulation acceleration

Machine learning offers an array of approaches for simulation acceleration. A common approach is to use machine learning in conjunction with ROM methods, where the learning algorithm augments or replaces part of the ROM method. Neural networks can be used to provide a powerful high-dimensional interpolation algorithm in the POD-Interpolation ROM approach [143,144,146] or to overcome the difficulties POD-Projection ROMs encounter for nonlinear equations [148,149]. Autoencoders can also replace the dimensionality reduction common across most ROM methods [34,35]. Another approach is to build a machine learning ROM based on autoencoders and feedforward neural networks while using POD for dimensionality reduction of the data passed to the machine learning ROM [145,153,154]. Machine learning can overcome some of the limitations of traditional ROMs and broaden the scope of problems for which the ROM methods are suitable.

PINNs are a machine learning-based simulation method that lies at the intersection of equation-based and data-driven modelling [36]. To be used for simulation acceleration, PINNs need to be able to generalise across new input parameters and/or geometries or they need to be sufficiently fast to train that a new PINN can be constructed for each new problem instance. The former can be achieved by adding extra inputs to the network or by constructing a precursor network that handles the parametric dependence in the problem [160,166,167]. Faster training times can be achieved through techniques such as transfer learning, trainable activation functions and residual-based adaptive refinement [163,168,170,171]. When used in an acceleration context, such as many-query parameter sweeps, PINNs have been demonstrated to reduce total simulation time by two to five orders of magnitude, depending upon the application and the number of queries [160,161]. PINNs and their extensions are suitable for all of the complexities that commonly occur in vascular flow problems and have been successfully applied to aneurysm flow modelling and synthesis of non-invasive flow measurements in a bifurcating vessel model [163,179].

Alternative machine learning-based simulation techniques include physics-agnostic methods, Point-Nets and operator networks. Physics-agnostic simulation methods have been applied to vascular flow problems such as fractional flow rate prediction in coronary arteries, steady-state pressure and velocity prediction in the thoracic aorta, inverse geometry prediction in the aorta, endothelial cell activation potential prediction and prediction of flow fields from magnetic resonance images [37,155,156,189,194]. While these approaches can accelerate solution evaluations by two to three orders of magnitude and tend to generalise well to previously unseen geometries, they require large datasets and the network outputs do not necessarily respect the underlying physics in the problem. Point-Nets facilitate the use of powerful convolutional deep learning architectures on datasets consisting of point clouds. They have been used for steady-state haemodynamics predictions before and after coronary artery bypass surgery and aneurysm flow diversion, producing accurate predictions and reducing prediction time by two to three orders of magnitude compared to the computational fluid dynamics model [38,39]. Point-Nets generalise well to new geometries despite paying no attention to underlying governing equations, but require large datasets for training. Physics can inform Point-Nets, but this is a new technique with very few use cases to date [199]. Operator learning techniques, such as DeepONets, are other powerful simulation techniques that have demonstrated strong generalisation capabilities, the ability to accelerate by two to five orders of magnitude, and the ability to overcome the curse of dimensionality [40,187,188]. However, operator learning is an emerging technique that has only seen a small number of applications to vascular flow problems to date [192,206].

### 4.2. Challenges

Despite years of research on ROMs and the recent application of machine learning to simulation acceleration, applying these techniques to real-world vascular flow problems remains challenging. Three key challenges to address that have been identified by this review are

1. The development of accelerated simulation methods that can handle large geometric variability, facilitating their application to previously unsimulated and dynamically varying geometries.
2. The development of accelerated simulation methods for multi-scale problems, enabling seamless evaluation of small- and large-scale processes over short- and long-term time scales.
3. The development of a benchmarking framework for accelerated simulation methods, allowing for systematic quantification and comparison of new approaches and driving transparent progress in the field.

A critical challenge to widespread adoption of simulation acceleration in vascular flow applications is incorporating large geometric variability into the models. Whether performing large-scale testing of medical devices in cohorts with varying anatomy, simulating medical device responses as part of treatment planning for an individual patient, or providing real-time surgical feedback during operation, the ability of the accelerated model to accurately evaluate haemodynamics in a previously unsimulated or dynamically changing geometries is essential. Efforts to introduce geometric variability into vascular flow ROMs have mainly focused on developing parameterised models [30,82,92,100]. While these approaches yielded accurate results, acceleration was only of one order of magnitude in most cases, with the largest acceleration roughly three orders of magnitude. Furthermore, models typically only used a small number of parameters describing features such as vessel diameter or stenosis severity and position [30]. In pathologies with highly complex shapes, such as aneurysms, identifying descriptive parameterisations with few parameters may not be possible. This would be further exacerbated by device modelling or fluid–structure interaction. A possible approach to overcome this is to use domain decomposition ROMs that can partition an unseen geometry into sub-geometries that bear resemblance to the geometries for which snapshots were previously calculated [208,209]. This approach has been applied to flow over urban landscapes and pipe flow problems so far, but could potentially be applied to vascular flow problems, where the sub-geometries could be a set of commonly required vascular segments and configurations. ML approaches such as physics-agnostic simulation methods [156,189,191] and Point-Nets [38,39] have demonstrated the ability to generalise to unseen geometries by using large sets of mostly synthetic geometries and corresponding simulation data for training. These are the most promising attempts to provide generalisation across geometries in vascular simulation acceleration, but they are still hampered by the amount of data required and the risk that data augmentation strategies can lead to unrealistic results. Informing these approaches with physics could potentially reduce the data requirement and increase the reliability of the results but there have been few studies into this to date [199].

Multi-scale problems represent the second challenge for accelerated simulation of vascular flow models. When using computational models to inform treatment decisions or in assessing medical device safety and efficacy, short- and long-term metrics are likely to be required. Depending upon the specific problem, models of small-scale processes like thrombosis or endothelialisation may need to be coupled to models of large-scale haemodynamic effects. In principle, DMD ROMs are well suited to long-term solution evaluation, but the few studies using this approach for vascular flow applications have focused on solution reconstruction rather than long-term prediction [105,109]. Domain decomposition PINN methods, such as cPINNs, XPINNs and PPINNs, are suitable for multi-scale problems in time and space, but also have seen little use in vascular flow applications [183–185]. DeepONets have also shown great potential for multi-scale applications. Wang & Perdikaris [205] used DeepONets for long-time prediction of partial differential equations, while Cai *et al.* [187] and Mao *et al.* [188] used modular DeepONets trained individually on single-physics single-scale problems to facilitate multi-physics and multi-scale modelling for electroconvection and flow-chemistry applications. Modular DeepONets are referred to as DeepM&MNets (Deep Multi-Physics & Multi-Scale Networks) and represent a promising approach towards the challenge of long-time evaluation of multi-physics and multi-scale models which are crucial in vascular flow applications.

The final challenge we want to highlight is the need for a benchmarking framework for assessing simulation acceleration methods. Throughout this review, quantitatively comparing different approaches has proved challenging due to the following factors that vary across studies: (i) amount of training data; (ii) training details, e.g. stopping/convergence criteria, number of modes retained in model; (iii) accuracy and acceleration metrics, e.g. error metrics and variables of interest, acceleration relative to FOM or entire offline cost; (iv) target applications. To overcome this challenge, we propose the development of a benchmarking framework for use in the simulation acceleration community. This should consist of a series of example problems of varying nature and complexity, datasets for each example problem for use in training, specified allowances and/or metrics for the computational cost of data generation and training, and metrics defined for assessment of accuracy and acceleration. The example problems should also be motivated by real-world problems where a balance often must be struck between the amount of training data available for the machine learning model and the task for which it is to be used (e.g. many-query tasks, control problems, real-time prediction etc.). Development and subsequent use of this framework would enable objective assessment and comparison of methodological advances in the field. Inspiration could also be taken from the medical image analysis field, where challenge problems are commonly proposed with publicly available data and predefined metrics to assess model performance for tasks like registration and segmentation [210,211].

### 4.3. Outlook

Accelerated vascular flow models are essential for applications such as *in silico* trials (ISTs), patient-specific treatment planning, and real-time surgery feedback. ISTs can require the evaluation of nonlinear, multi-physics, multi-scale models in large cohorts of virtual patients, which are anatomically and physiologically diverse, undergoing treatment with different devices [3,212]. Patient-specific treatment planning requires similarly complex models that can be evaluated in an individual patient in a reasonable time frame given the prognosis of the pathology in question. Real-time surgery feedback requires complex model evaluation in individual patients fast enough to provide haptic feedback or visualisations to the surgeon performing the procedure [44]. These three applications highlight some of the impact that accurate and efficient vascular flow models can have on patient care, which makes developing these approaches a worthwhile endeavour. This review has identified that the key challenge to be addressed is the development of multi-scale simulation acceleration methods that can handle the large geometric variability inherent to vascular flow problems. We also suggest that to achieve quantifiable and transparent progress in simulation acceleration, the community should develop a benchmarking framework consisting of a series of exemplar problems with standardised metrics for assessing acceleration and accuracy. This would benefit both the simulation acceleration and the vascular flow modelling communities.

## Data Availability

This article has no additional data.
